# Probing Allosteric Hsp70 Inhibitors by Molecular Modelling Studies to Expedite the Development of Novel Combined F508del CFTR Modulators

**DOI:** 10.3390/ph14121296

**Published:** 2021-12-12

**Authors:** Roberto Sabbadini, Emanuela Pesce, Alice Parodi, Eleonora Mustorgi, Santina Bruzzone, Nicoletta Pedemonte, Monica Casale, Enrico Millo, Elena Cichero

**Affiliations:** 1 Department of Pharmacy, Section of Medicinal Chemistry, School of Medical and Pharmaceutical Sciences, University of Genoa, Viale Benedetto XV, 3, 16132 Genoa, Italy; sabbadove96@icloud.com; 2 UOC Genetica Medica, IRCCS Istituto Giannina Gaslini, Via Gerolamo Gaslini, 5, 16147 Genova, Italy; emyfish85@yahoo.it (E.P.); nicolettapedemonte@gaslini.org (N.P.); 3 Department of Experimental Medicine, Section of Biochemistry, University of Genoa, Viale Benedetto XV 1, 16132 Genoa, Italy; alice.parodi1994@gmail.com (A.P.); santina.bruzzone@unige.it (S.B.); 4 Department of Pharmacy, Section of Chemistry and Food and Pharmaceutical Technologies, University of Genoa, Viale Cembrano, 4, 16148 Genoa, Italy; mustorgi@difar.unige.it (E.M.); casale@difar.unige.it (M.C.)

**Keywords:** HSP70, allosteric inhibitor, MKT-077, CFTR modulator, correctors, virtual screening, QSAR

## Abstract

Cystic fibrosis (CF) is caused by different mutations related to the cystic fibrosis transmembrane regulator protein (CFTR), with F508del being the most common. Pioneering the development of CFTR modulators, thanks to the development of effective correctors or potentiators, more recent studies deeply encouraged the administration of triple combination therapeutics. However, combinations of molecules interacting with other proteins involved in functionality of the CFTR channel recently arose as a promising approach to address a large rescue of F508del-CFTR. In this context, the design of compounds properly targeting the molecular chaperone Hsp70, such as the allosteric inhibitor MKT-077, proved to be effective for the development of indirect CFTR modulators, endowed with ability to amplify the accumulation of the rescued protein. Herein we performed structure-based studies of a number of allosteric HSP70 inhibitors, considering the recent X-ray crystallographic structure of the human enzyme. This allowed us to point out the main interaction supporting the binding mode of MKT-077, as well as of the related analogues. In particular, cation-π and π–π stacking with the conserve residue Tyr175 deeply stabilized inhibitor binding at the HSP70 cavity. Molecular docking studies had been followed by QSAR analysis and then by virtual screening of aminoaryl thiazoles (**I**–**IIIa**) as putative HSP70 inhibitors. Their effectiveness as CFTR modulators has been verified by biological assays, in combination with VX-809, whose positive results confirmed the reliability of the whole applied computational method. Along with this, the “in-silico” prediction of absorption, distribution, metabolism, and excretion (ADME) properties highlighted, once more, that AATs may represent a chemical class to be further investigated for the rational design of novel combination of compounds for CF treatment.

## 1. Introduction

Cystic fibrosis (CF) is the autosomal recessive disorder most common in Caucasian populations [1]. It is caused by different mutations in the cystic fibrosis transmembrane regulator protein (CFTR) [2,3].

This protein belongs to the ABC (ATP-Binding Cassette) Transporter Superfamily, being made of five distinctive domains, namely two transmembrane domains (TMDs), two nucleotide-binding domains (NBDs), and a regulatory (R) domain. It is well established that CFTR’s R domain needs to be phosphorylated by protein kinase A (PKA) before the channel can be gated by ATP [4]. In fact, the TMDs form the channel across the membrane, and the NBDs bind and hydrolyze ATP to stimulate opening and closing of the channel, regulated by (de)phosphorylation, which changes the interactions of R [5,6,7].

More than 2500 mutations of the CFTR gene are known [3]. The most common mutation, F508del, belongs to the class II trafficking defects, where folding of the CFTR protein is impaired, resulting in a reduction of the amount of ion channels present on the cell surface. Although CF is a systemic disease, the most severe effects come from the reduced chloride secretion in the airways. Subsequent lung surface dehydration leads to the buildup of a thick mucus layer, which clogs the airways and traps bacteria, leading to infections, extensive lung damage, and, eventually, respiratory failure [8,9].

Most treatments, such as antibiotics and mucus-clearing agents, are symptomatic; hence, there is a great need for disease-modifying agents [10,11]. It is believed that the folding and channel activity defect of F508del can be addressed by means of modulators named correctors and potentiators, respectively [12,13,14,15,16].

CFTR correctors (e.g., lumacaftor; VX-809) [17] allow for the transfer the mutant proteins to the plasma membrane, while CFTR potentiators (e.g., ivacaftor; VX-770) [15] strongly increase channel gating. Since neither lumacaftor nor ivacaftor, by themselves, seem to have clinical benefits for CF patients with the F508del mutation, [18,19] combination therapy with the two drugs has been applied. This approach improved lung function and disease stability [20], leading to the approval of lumacaftor–ivacaftor combination therapy, such as Orkambi [21,22,23], healing lung function to a modest degree in F508del homozygous patients. On the other hand, triple combinations, using two distinct correctors and a potentiator, showed good clinical benefit in patients with a F508del-CFTR mutation on at least one allele [24,25,26,27]. Recently, elexacaftor was found able to multiplicative synergy with the ivacaftor in rescuing multiple CFTR class defects, supporting the effectiveness of new therapeutic combination of these compounds for CF therapies. This agreed with their incorporation into a triple combination therapeutic (marketed as Trikafta) in presence of the corrector tezacaftor [28].

While the effectiveness of these combined drugs, through the multitude of genotypes, remains to be evaluated, combinations of molecules interacting with other proteins involved in the functionality of the CFTR channel are expected to result in a large rescue of F508del-CFTR [29,30].

In this context, the design of compounds properly targeting the molecular chaperone Hsp70 have recently been discussed as a promising strategy to optimize drug combinations for the treatment of CF [31,32]. Indeed, molecular chaperones assist the folding and assembly of the cytosolic domains of CFTR, with Hsc70 (HSPA8), its inducible homolog Hsp70 (HSPA1A/B), and Hsp90 being the most important [33,34].

Recent studies, reported in the literature by Young and co-workers [31], pointed out a key role played by allosteric ligands targeting the Hsc70/Hsp70 proteins to deeply amplify the corrector ability of lumacaftor. In particular, they shown that the stability barrier of rescued F508del-CFTR can be circumvented by a new combination of mechanisms. While VX809 corrects the trafficking defect of F508del CFTR, allosteric inhibitors of HSP70, such as MKT-077, allow the further accumulation of the rescued protein. MKT-077 increased the stability of F508del CFTR against degradation at the endoplasmic reticulum, relieving the suppression of maturation by Hsc70/Hsp70 over longer time scales [31].

Since MKT-077 can affect Hsc70/Hsp70-mediated degradation of CFTR both during and after maturation, it is believed that may also be effective against other misfolding CF mutations, such as R560T, A561E, R1066C, and N1303K. This opens a promising scenario for the development of effective therapies to be used to contrast other CF mutations.

MKT-077 is a cationic rhodacyanine, proposed as an anti-proliferative agent, active against MCF-7 breast cancer cells. Despite its little cytotoxicity against either normal human fibroblasts or immortalized epithelial cells, it was withdrawn by Phase I clinical trials because of poor metabolic stability [35]. For this reason, during the last years, a number of studies have been performed to modify the chemical structure of the prototype towards novel compounds maintaining the same HSP70 allosteric modulator ability but exhibiting a better safety and pharmacokinetic (PK) profile [36,37].

In this context, we deemed it interesting to better explore the putative binding mode of a consistent series of MKT-077 analogues that have, so far, been reported in the literature [36,37] as allosteric HSP70 inhibitors, exhibiting variable ability to inhibit cellular growth in cancer lines (MKT-077, YM-01 and **1**–**90**; see Supplementary Tables S1 and S2). Indeed, it has been found that the MCF-7 antiproliferative ability of these series of compounds was related to the HSP70 inhibitory one, by testing the ligand binding affinity towards the HSP70 protein of active/inactive ligands, when evaluated as MCF-7 antiproliferative agents [37].

**Figure 1:**
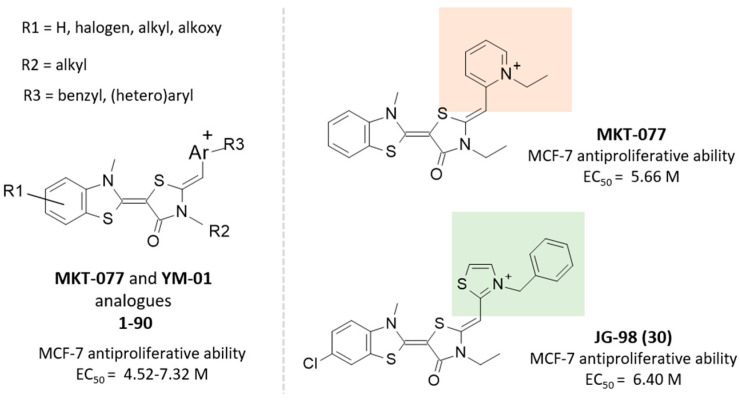
Chemical structure and EC_50_ values of different MKT-077 analogues, biologically evaluated as MCF-7 antiproliferative agents. The most exploited (hetero)cyclic rings in the search of novel, promising HSP70 inhibitors are highlighted in orange and green.

As shown in Figure 1, most of the collected compounds maintained the MKT-077 pyridinium substituent as a terminal group, while a number of them have been optimized as thiazolyl-containing congeners, as experienced by JG-98 (**30**) and JG-231 (**71**).

Herein, their most important key features involved in the MCF-7 antiproliferative ability as a result of its activity as an HSP70 inhibitor have been analyzed performing molecular docking studies and Quantitative-Structure Activity Relationship (QSAR) analyses.

In particular, while the docking calculations so far reported in the literature were performed based on the bovine protein, the recent X-ray crystallographic of the human HSP70 data released on the protein data bank (pdb code: 6CZ1 released: 10 April 2019, resolution = 1.68 Å) [38] allowed us to apply a more reliable structure-based approach, towards the design of further new molecules.

These findings paved the way for the following screening and evaluation of novel MKT-077 analogues, to be exploited, in combination with F508del correctors, conceivably in order to improve their rescue ability. This was done by initially focusing on the benzothiazole-containing compounds present in our in-house series of aminoarylthiazoles (AATs), previously explored as F508del-CFTR correctors [39].

Indeed, our research group synthesized a number of thiazole-based compounds with an interesting ability to improve both the trafficking and gating of mutant CFTR [40,41], with a lot of them a library of new AAT-VX-809 hybrid derivatives. These compounds proved to act, especially, as correctors in biochemical experiments [40,41].

Herein, MKT-077 and the best-scored and filtered AATs **I**–**IIIa** have been evaluated, in combination with VX-809, in order to confirm their CFTR modulator effectiveness. Meantime, the insilico prediction of descriptors, related to absorption, distribution, metabolism, and excretion properties (ADME), have been determined, in order prioritize the most promising AATs for further optimization. This approach allowed us to assess the rescue ability of the selected multi-compound treatment, supporting the CFTR amplifier ability promoted for the allosteric HSP70 inhibitors.

In addition, the explored **I**–**IIIa**, which may represent lead compounds for the development of optimized multi-drugs that correct the basic defect in CF patients, being of high relevance for the development of treatments able to revert or arrest the progression of the disease.

## 2. Results and Discussion

### 2.1. Exploring In Silico the Human HSP70 Protein

Up to now, structure-based studies concerning the putative binding positioning experienced by the well-known HSP70 inhibitor MKT-077 have performed, taking into account the X-ray data of the bovine protein (PDB code = 3HSC) [42]. These studies revealed that MKT-077 and its analogs bind within a highly conserved, hydrophobic pocket, adjacent to the nucleotide-binding cleft. In particular, the main benzothiazole core shows favorable hydrophobic contacts with the protein cavity, while the rhodacyanine group appears to play a role in orienting the other rings but detecting a few numbers of direct contacts. Conversely, the benzothiazole ring occupied a relatively narrow pocket, suggesting the need of a flat core able to occupy this region, showing small substituents.

**Figure 2:**
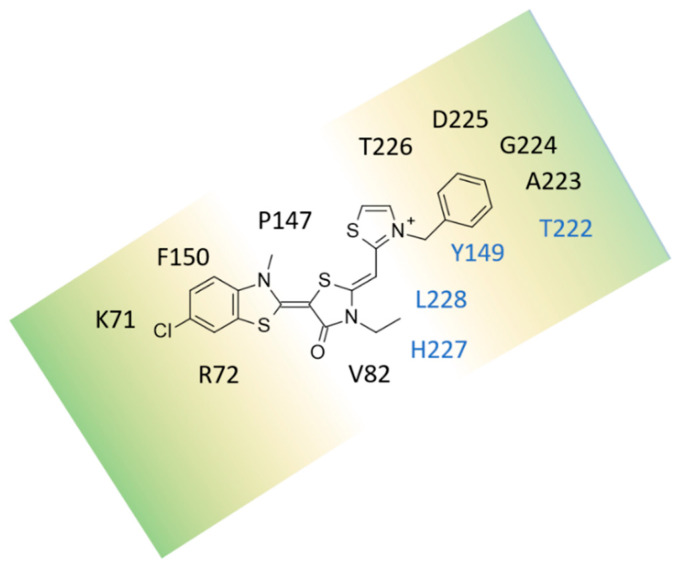
Schematic representation of the main contacts involving the MKT-077 and bovine HSP70, as described in the literature. The key residues for the ligand binding, as shown by mutagenesis experiments, are labelled in cyan [37].

In particular, according to the studies reported by Gestwicki [36,37,43,44], the HSP70 allosteric inhibitors, such as MKT-077 and the analogue JG-98 (**30**), occupy the enzyme cavity, delimited by K71, R72, P147, and F150, thanks to the flat benzothiazole core, while the terminal cation portion protruded towards T222, A223, G224, D225, and T226 (Figure 2). The two tethering five-membered rings were surrounded by V82, Y149, H227, and L228.

Based on the cellular potency, as antiproliferative agents, evaluated in MCF-7 cells, featured by the prototype MKT-077 (pEC_50_ = 5.66 M), and more potent congeners JG-98 (**30**; pEC_50_ = 6.40 M) and JG-294 (**89**; pEC_50_ = 7.00 M), the same authors evaluated the affinity JG-294, as representative of the series for purified Hsp70 in vitro, using a fluorescence shift assay. Compound JG-258 (**62**; pEC_50_ = 5.30 M) was also used as negative control.

They identified that JG-294 (**89**) displayed an affinity value of 0.35 μM, while JG-258 (**62**) did not bind (Ki > 10 μM). Following mutagenesis experiments, involving the Y149W, T222A, H227A, or L228A mutants, confirmed the key role, played by the four mentioned residues, since each mutation reduced the affinity of JG-294 (**89**) by 4- to 20-fold [37].

Herein, we proceed for the first time to a careful analysis of the docking mode of the inhibitor MKT-077, as well as of the analogues reported in the literature, see Supplementary Tables S1 and S2), relying on the experimental data of the human enzyme (PDB code = 6CZ1) [37]. This kind of approach is expected to clarify the most important chemical interactions, and the related amino acids supporting the compound HSP70 inhibitory ability, and to open the way for the following virtual screening strategy.

**Figure 3:**
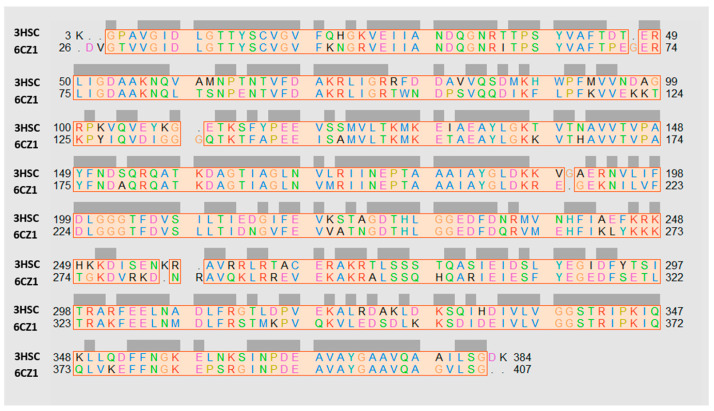
Sequence alignment of the human HSP70 (PDB code = 6CZ1) [37], with respect to the bovine one (PDB code = 3HSC) [42]. The conserved residues are highlighted in grey. Poorly conserved aminoacids, in terms of hydrophilic or hydrophobic properties, are shown in black. When polar features or lipophilic requirements are shared or maintained, the related couple of aligned residues is colored. The alignment was depicted thanks to the visualization UCSF Chimera software [46].

Initially, the three-dimensional coordinates of the human Hsp70 (monomer A) and bovine one had been aligned and superposed, by means of MOE software [45], giving pairwise percentage residue identity values of 69.9%, while the root mean square deviation value (RMSD; calculated on the carbon atom alignment) between the two proteins was of 1.254 Å (see the protein alignment in Figure 3).

**Figure 4:**
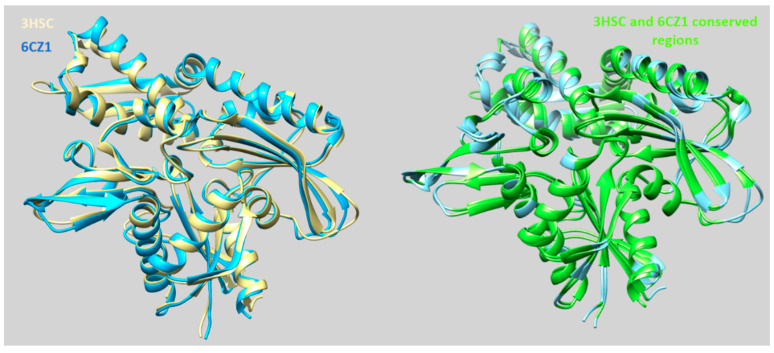
Superimposition of the X-ray crystallographic data of the human HSP70 (PDB code = 6CZ1; ribbon in cyan) [37] onto those of the bovine protein (PDB code = 3HSC; ribbon in light yellow) [42] (**left** side). A perspective of the conserved regions of the two proteins, based on the visualization UCSF Chimera software [46], is highlighted in green (**right** side).

These findings supported a good similarity, shared by the two enzymes, as confirmed by the superimposition of 6CZ1 on the X-ray data of 3HSC, reported in Figure 4 (left; ribbon in cyan and light yellow, respectively). A perspective of the overall conserved regions of the two proteins is also shown in green (Figure 4; right).

Accordingly, several residues resulted to be maintained along the whole structure of the two Hsp70 proteins, suggesting quite comparable binding sites at the two macromolecules. As shown in Table 1, a number of residues supporting the MKT-077 binding at the bovine HSP70 cavity, as reported in the literature [37], are conserved at the human HSP70. Among them, four aminoacids, scouted as key residues for the ligand binding at the bovine protein by mutagenesis experiments (Y149, T222, H227, and L228), were maintained as Y175, T247, H252, and L263 within the human protein.

Nevertheless, two residues featured electrostatic and steric variation, moving from the bovine enzyme to the human one, as exemplified by the two small and hydrophobic A223 and V82 residues in 3HSC, which correspond to the human hydrophilic N248 and S107 residues. As a consequence, the overall cavity delimited at the 6CZ1 allosteric binding site could be differently occupied by the inhibitors.

### 2.2. Molecular Docking Studies of MKT-077 and Related Analogues

On this basis, and to further assess the reliability of the human HSP70 putative binding site, proposed as previously described, we have considered the site finder module of the MOE software to identify the best score binding site at 6CZ1. The results suggested and confirmed the same crevice, identified by comparison with 3HSC, giving us the opportunity to proceed with the following molecular docking calculations described.

Starting from this data, we performed docking studies of the reference compound MKT-077, as well as of the aforementioned different analogues reported in the literature **1–90** (see Supplementary Tables S1 and S2). The related scoring functions have been shown in Supplementary Table S3.

**Figure 5:**
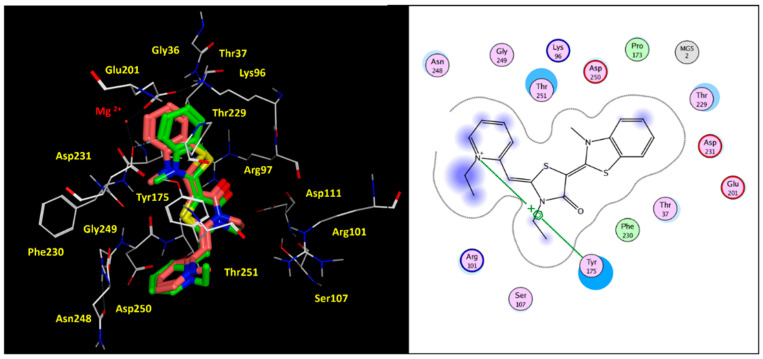
Docking positioning of MKT-077 (C atom; green) and analogue YM-01 (C atom; pink) within the modelled human HSP70 protein. The most relevant residues are shown in yellow (**left** side), while key contact involving the conserved residue Tyr175 is highlighted in the corresponding ligplot (**right** side).

Based on our calculations, the docking positioning of the reference Hsp70 allosteric inhibitor MKT-077 experienced polar contacts, involving the carbonyl oxygen atom of the main five-membered ring and side chains of R97 and T251 (see Figure 5). In addition, it proved to be highly stabilized at the HSP70 surface, thanks to: (i) polar and cation-π interactions, involving the terminal benzothiazole ring and Mg^2+^, K96; (ii) cation-π contacts between the pyridine-containing cation group and Y175 sidechain.

**Figure 6:**
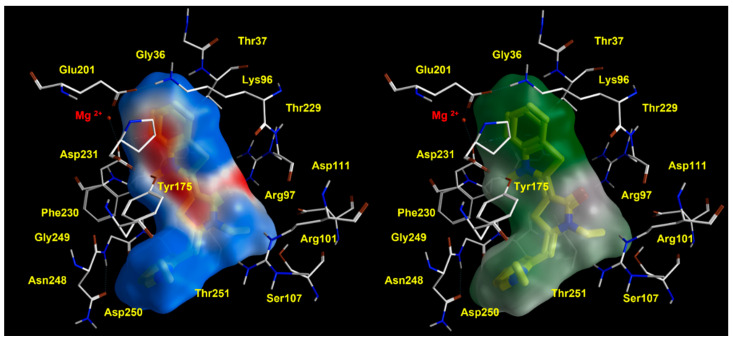
Electrostatic properties of the HSP70 inhibitor YM-08, shown on the related molecular surface (**left** side). The corresponding ligplot is reported on the **right**.

Notably, the key role played by interacting with Y175 was in accordance with mutagenesis experiments solved by other authors for the corresponding bovine HSP70 residue Y149 (see the previously cited Table 1). Thus, specific requirements, in terms of proper balanced hydrophilic and hydrophobic properties within the allosteric inhibitor, resulted to be mandatory to fit the protein surface, as shown in Figure S1, as a ligplot of YM-01. Accordingly, YM-01 featured an extended and consistent electron-rich flat core, which was properly projected towards positively charged motifs of the biological target, such as Mg2+, Lys96, and Arg97. Conversely, the inhibitor cation moiety was oriented in proximity of the solvent-exposed surface of the HSP70 protein, being involved in cation-π stacking with Tyr175 (see Figure 6, the electrostatic properties of YM-01 are shown on the left).

It is worth noting that the aforementioned electrostatic properties turned out to be efficiently guaranteed by the conjugated system, through the folded aromatic structure of the inhibitor YM-01, being endowed with two terminal aromatic rings. These would contribute to stabilize the compound at the HSP70 surface, thanks to aromatic interactions and cation-π stacking with Tyr175 and Lys96, also fulfilling the steric requirements, as described by this narrow pocket of the enzyme (see Figure 6, where the lipophilic properties of YM-01 are shown on the right).

With the introduction of several chemical substituents on two reference inhibitors MKT-077 (MCF-7 pEC_50_ = 5.66) and YM-01 (MCF-7 pEC_50_ = 5.28), and, in particular, with the insertion of halogens or ether substituents in the main bicyclic core, effective fluorinated analogues, such as **5**–**8** (MCF-7 pEC_50_ = 5.62–6.10) and **1**–**4** (MCF-7 pEC_50_ = 4.74–6.00), have been constructed, respectively.

In particular, choosing the 3-F group in R1 improved the potency of both **1** (MCF-7 pEC_50_ = 5.66) and **5** (MCF-7 pEC_50_ = 6.00), if compared to YM-01 and MKT-077.

**Figure 7:**
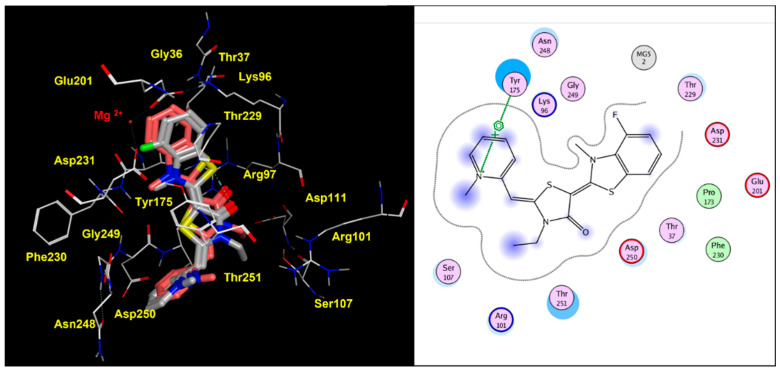
Docking positioning of **1** (C atom; gray) and of the prototype YM-01 (C atom; pink) within the modelled human HSP70 protein. The most relevant residues are shown in yellow (**left** side), while key contact involving the conserved residue Tyr175 is highlighted in the corresponding ligplot (**right** side).

As shown in Figure 7, the docking positioning observed for the HSP70 inhibitor **1** was highly comparable with that of YM-01, revealing a proper role played by the electron-rich 3-F group to detect polar contacts with Mg^2+^.

Any substitution to the substituents R2 and R3, considering the (aryl)alkyl groups, resulted in a promising effect, created by the benzyl group in R3 (rather than R2), by maintaining the ethyl part in R2, especially in the presence of the chlorine atom or additional hydrophobic groups in R1, at position 5 of the bicyclic nucleus.

Indeed, the 5-Cl and 5-methyl-substituted **30** (JG-98; MCF-7 pEC_50_ = 6.40) and **40** (JG-194; MCF-7 pEC_50_ = 6.80) were more potent than the corresponding 4-substituted analogues **29** (MCF-7 pEC_50_ = 6.22) and **39** (MCF-7 pEC_50_ = 6.68). Then, bearing in mind all the HSP70 inhibitors featuring the unsubstituted benzyl group as R3 (**20**, **26–48**; MCF-7 pEC_50_ = 5.30–7.10), compound **43** that exhibit the 5-ethyl substituent in R1 was the most effective (MCF-7 pEC_50_ = 7.10). The related docking positioning is shown in Figure S2.

These data highly motivated the further development of benzyl-substituted analogues bearing bulky groups at the same benzyl moiety. Among them, the *ortho*-benzyl-substituted analogues proved to be often more effective than the *meta* or *para*-substituted ones, as confirmed by the higher potency of **49** (MCF-7 pEC_50_ = 6.80) and **52** (MCF-7 pEC_50_ = 6.72), if compared to **50–51** (MCF-7 pEC_50_ = 6.21–6.32) and **53–54** (MCF-7 pEC_50_ = 5.84–6.59), respectively (see the chemical structures in Tables S1 and S2). In addition, the *ortho*-benzyl-substituted inhibitors **55–61** (MCF-7 pEC_50_ = 6.40–6.89) bearing the 5-Cl substituent onto the benzothiazole-based ring, were more potent than the unsubstituted analogue **30** (JG-98) (MCF-7 pEC_50_ = 6.40).

Interestingly, the presence of H-bond acceptor functions on the benzyl group proved to be beneficial, especially when alkyl chains or chlorine atoms were maintained at the main bicyclic core. Thus, the 5-CH_3_ and 5-OCH_3_ analogues **78** (MCF-7 pEC_50_ = 7.19) and **80** (MCF-7 pEC_50_ = 7.15), exhibiting the 2-OCF_3_-benzyl ring in R3, were more potent than **40** (JG-194) (MCF-7 pEC_50_ = 6.80) and **45** (MCF-7 pEC_50_ = 6.89).

Accordingly, the 5-CH_2_CH_3_ congener **86** (JG-345) (MCF-7 pEC_50_ = 7.31) displayed higher pEC_50_ than **43** (MCF-7 pEC_50_ = 7.10), being one of the most effective MKT-077 analogue.

**Figure 8:**
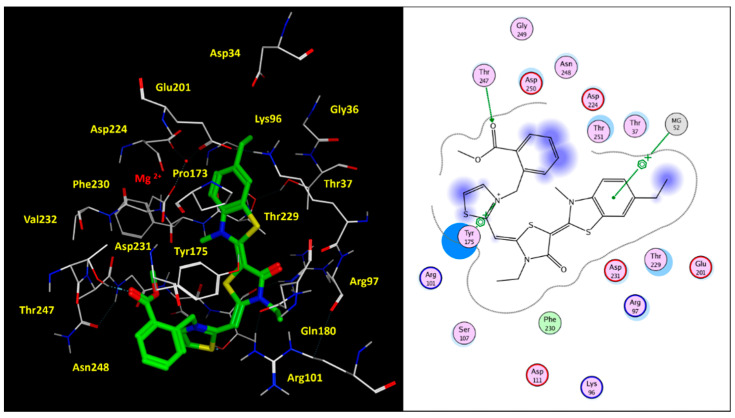
Docking positioning of **86** (C atom; green) within the modelled human HSP70 protein. The most relevant residues are shown in yellow (**left** side), while the key contact involving the conserved residue Tyr175 and Thr247 are highlighted in the corresponding ligplot (**right** side).

Indeed, as shown in Figure 8, the benzothiazole moiety and terminal benzyl group were projected towards the cation Mg^2+^ and the Tyr175, displaying cation-π and π–π stackings. This kind of positioning was deeply stabilized by additional H-bonds, involving the ester moiety and Thr247, while the carbonyl oxygen atom and protonated nitrogen one experienced polar contacts with the surrounding Arg97 and Asp250.

Moreover, the isosteric replacement of the benzyl group as R3 with the thiophene ring led to further MKT-077 analogues, such as **68–72** (MCF-7 pEC_50_ = 6.14–7.01), also endowed with MCF-7 inhibitory ability, including some with comparable potency values, compared to benzyl-containing compound **30** (MCF-7 pEC_50_ = 6.40).

It is thought that proper steric properties are required for optimized aromatic substituents as R3, which would efficiently fit the enzyme cavity delimited by Tyr175, Asn248, Gly249, Asp250, and Thr251. This information is supported by the high pEC_50_ value observed for the dimethyl-substituted thiophene-containing derivative **72** (MCF-7 pEC_50_ = 7.01).

### 2.3. QSAR Analyses

During the last years, applying computational methods to the search of novel bioactive compounds proved to be highly effective and useful along the drug discovery process [47,48]. While protein modelling allows to simulate the main drug-target binding interactions, QSAR analyses turns in quantitative methods to predict the potency of further novel compounds [49,50].

In this context, we deemed it interesting to explore, by development of a QSAR model, the different potency featured by the MKT-077 analogues, herein investigated (**1–90**) as putative HSP70 inhibitors, referring to their MCF-7 antiproliferative ability.

The QSAR model, developed considering the compound positioning observed by docking calculations, is expected to deepen our knowledge of the main relevant chemical substitutions to efficiently design novel MKT-077 analogues as MCF-7 inhibitors.

With this aim, about three hundred molecular descriptors (including 2D and 3D parameters) were estimated, thanks to MOE software [45]. The bidimensional parameters are classified in seven subsets, including physical properties (2D-I), subdivided surface areas (2D-II), atom and bond counts (2D-III), connectivity-based descriptors (2D-IV), partial charges descriptors (2D-V), pharmacophore features descriptors (2D-VI), and the so-called adjacency and distance matrix descriptors (2D-VII). The 3D-descriptors are divided in five groups, such as the potential energy (3D-I), and MOPAC (3D-II), surface area (3D-III), volume and shape (3D-IV), and conformation-dependent charge descriptors (3D-V).

This study was performed applying a statistical approach, previously described [51].

The final model was obtained by splitting the compounds into a training and test set, using the Kennard–Stone design [52], one of the most popular algorithms for guiding the selection of a subset of samples with a distribution as close as possible to the uniform distribution. In particular, the Kennard–Stone algorithm was performed, adding the response vector (pEC_50_) as an additional column to the matrix of the descriptors, in order to ensure that the training set compounds were distributed evenly within not only in the space defined by the descriptors but also by the response values [53].

Among the 333 molecular descriptors, the most informative ones were identified using a multivariate variable selection method. In particular, iterative stepwise elimination PLS (ISEPLS) [54] was applied, in order to evaluate the relevance of the predictors, with regard to the possibility of predicting the response variable y (pEC_50_). Following this approach, eight descriptors were retained, as related to the MCF-7 inhibitory ability experienced by the collected compounds, most of them being 2D descriptors (see Table 2). Indeed, five descriptors fall in the 2D cluster, as follows: one in the 2D-I group, two in the 2D-II group, and one in the 2D-V and 2D-VI groups, respectively. Then, two parameters fall in the 3D-I cluster, with the final one in the 3D-VI.

The predictive model was calculated by dividing the whole dataset of ninety-two compounds into a training set, (MKT-077, **1**, **3–12**, **14**, **16–19**, **21–25**, **27**, **28**, **30**, **31–34**, **36**, **38**, **41–45**, **47**, **48**, **50–54**, **57–62**, **63–66**, **68**, **69**, **71–76**, **78–81**, **83–85**, **87–90**) to develop the QSAR model, and into a test set, including twenty derivatives (YM-01, **2**, **13**, **15**, **20**, **26**, **29**, **35**, **37**, **39**, **40**, **46**, **49**, **55**, **56**, **67**, **70**, **77**, **82**, **86**), in order to assess the reliability of the regression model. In particular, the final model was generated by employing PLS analysis to give a cross validated r^2^ (r^2^_cv_) = 0.78, a non-cross validated r^2^ (r^2^_ncv_) = 0.82, root mean square error (RMSE) = 0.310, and a test set r^2^ (r^2^_pred_) = 0.85. The predicted and experimental MCF-7 inhibitor ability for all the compounds is reported as tables, along with the collected descriptors, as shown in Supplementary Table S4.

**Figure 9:**
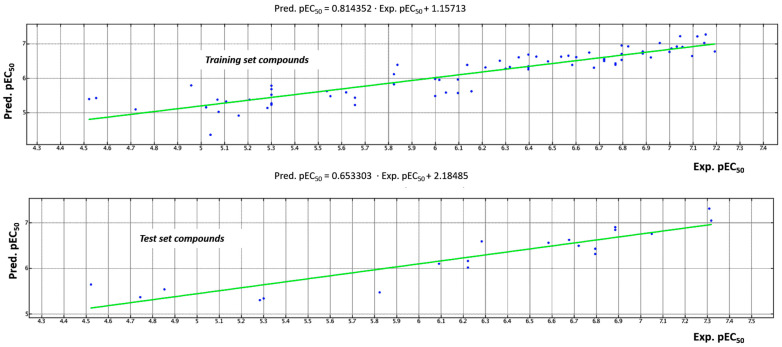
Distribution of the predicted (Pred.pEC_50_) and experimental (Exp.pEC_50_) potency values of the training set and test set ligands (by blue circles).

A perspective of the high performance of this model is represented in Figure 9, in terms of distribution of the predicted (Pred.pEC_50_), with respect to the experimental (Exp.pEC_50_) potency values of the dataset compounds.

Quantitatively, the MCF-7 inhibitory ability of the compounds, here investigated, is explained by the following Equation (1):

pEC_50_ = 2.30164 + 0.06220 · bpol − 0.11591 · E_str + 0.03423 · E_strain + 0.00122 · Q_VSA_POS − 0.03745 · SlogP_VSA1 − 0.01321 · SlogP_VSA3 + 0.00554 · vsa_hyd + 0.04307 · vsurf_DD23 (1)

Most of the selected descriptors deal with the polarity profile of the compounds (such as bpol and Q_VSA_POS), extended along the molecular surface of the ligands, which would exhibit quite flat or poorly flexible conformation (see the E_str descriptor). This turns in specific requirements, in order to guarantee the necessary hydrophilic/hydrophobic properties onto the overall surface extent, as suggested by the vsa_hyd descriptor. This is in harmony with the results obtained by docking calculations, regarding the likely role played by two aromatic terminal rings being engaged in cation-π or π–π stacking with the cation Mg^2+^ and Tyr175.

In particular, in Table 3, the mean values of a number of descriptors chosen as representative for the related 2D and 3D sub-classes are reported based on the main cation portion of the compounds. Only the most active thiazole-containing derivatives (pEC_50_ > 6.80 M) had been considered as references for optimized derivatives.

Among them, the E_str and SlogP_VSA1 proved to be negatively related to the compound potency (shown in italic in Table 3), while increasing values of bpol, Q_VSA_POS, and Vsa_hyd led to more promising analogues. Accordingly, the calculated mean values of E_str for the most potent thiazoles (pEC_50_ > 6.80 M; E_Str _mean_ = 3.5747) was lower than that for the less promising pyridine-based analogues (pEC_50_ 4.72–6.10; E_Str _mean_ = 18.4938), while the related SlogP_VSA1 mean values were quite comparable. Then, all the parameters positively related to the biological activity reported in Table 3 (bpol, Q_VSA_POS and Vsa_hyd) were properly higher for the most promising thiazoles than for the bioisostere pyridine-containing precursors.

**Figure 10:**
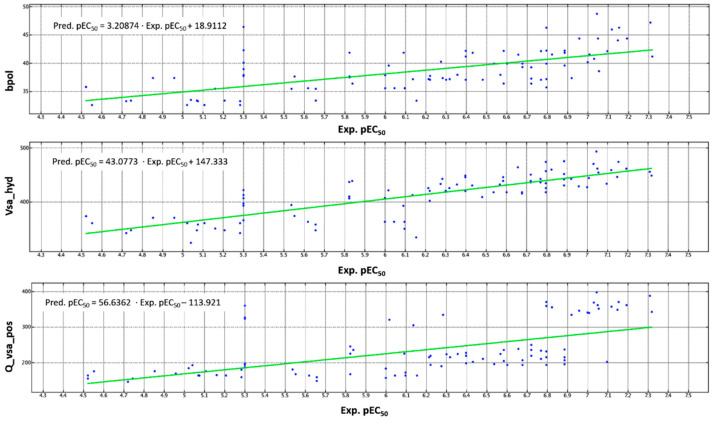
Distribution of the bpol (**up**), Vsa_hyd (**middle**), and Q Vsa_Pos (**down**) descriptor values, with respect to the experimental (Exp.pEC_50_) potency data of the whole dataset compounds (shown as blue dots).

A schematic representation of the role played by most of them is shown in Figure 10, once again revealing the high correlation between the reported descriptors and experimental pEC_50_ values of the whole dataset.

Along with this information, the optimal number of the bpol, Q_VSA_POS, and Vsa_hyd descriptors should be higher than 40, 300, and 400, respectively.

The reliability of this information is verified by the mean values observed for the aforementioned bpol, Vsa_hyd, and Q_VSA_POS, when focusing on the only most active thiazoles (pEC_50_ > 6.80 M). As shown in Table 4, within the thiazole series, the most potent benzyl-containing derivatives featured slightly higher values for these parameters than the five-membered ring analogues, with the exception of Q_VSA_POS. On the other hand, the five-membered, ring-containing analogues displayed limited, just adequate bpol values.

Despite this, by an overall perspective of the data shown in Table 4, in any case, the described thiazoles experienced acceptable descriptor values, often in accordance with the previously recommended ones (bpol > 40, Q_VSA_POS >300 and Vsa_hyd > 400).

Indeed, it is worth noting that one of the most potent five-membered, ring-based MKT-077 analogues **72** (pEC_50_ = 7.01; bpol = 41.5818, Q_VSA_POS= 340.5153, and Vsa_hyd = 444.6678) was endowed by comparable parameter levels, with respect to **86** (pEC_50_ = 7.31; bpol = 47.2218, Q_VSA_POS = 388.8322, and Vsa_hyd = 456.3374), which is one of the most effective MKT-077 analogues of the whole dataset.

### 2.4. Virtual Screening of AATs as Putative HSP70 Inhibitors

In the search of CFTR modulators, during the last years, we evaluated various AATs with the interesting ability to improve both the trafficking and gating of mutant CFTR [39], while, more recently, we have synthetized a library of new AAT-VX-809 hybrid derivatives featuring the reference corrector benzodioxole-based carboxamide moiety, maintaining the prototype corrector ability [40,41].

Thus, this novel class of correctors was further optimized by us towards the discovery of a number of analogues featuring comparable potency, with respect to VX809 (EC_50_ = 2600 nM), such as compound **7m** (EC_50_ = 70 nM), shown in Figure S3. The effectiveness of these compounds, as F508del CFTR correctors, was confirmed by biological and biochemical experiments [41].

Nowadays, based on the efficient F508del-CFTR rescue, achieved by co-administration of VX809, in the presence of the HSP70 allosteric inhibitor MKT-077 [31], we proceeded with virtual screening campaigns towards the discovery of novel likely HSP70 inhibitors.

**Figure 11:**
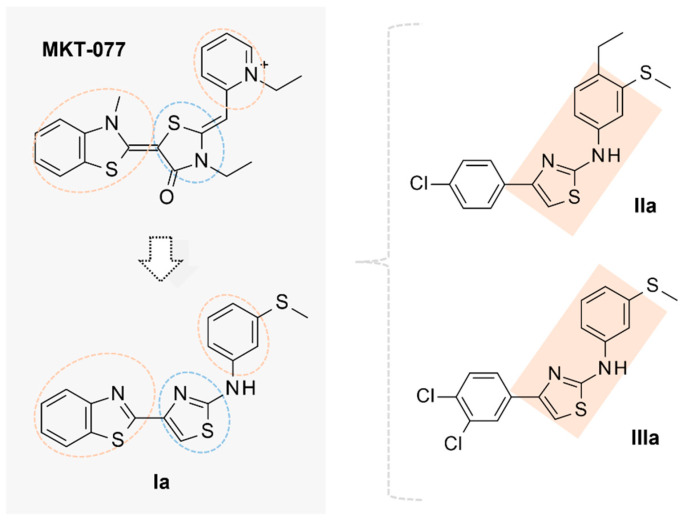
A comparison of the chemical features displayed by **Ia**, with respect to the allosteric HSP70 prototype MKT-077, being the two terminal aromatic motifs and central H-bonding core, highlighted by orange and blue dotted circles, respectively (**left**). The chemical structures of the two **Ia** analogues (**II**–**IIIa**) are also shown, being the maintained amino-aryl thiazole core of **Ia** highlighted in orange (**right**).

Based on the aforementioned information, obtained by molecular docking studies and QSAR calculations, we filtered a number of MKT-077 analogues within the in-house series of AATs, taking into account their ability to share the observed docking mode of the reference compound MKT-077. In particular, we focused on and then better explored compound **Ia**, whose chemical structure highly resembles the one of the prototype MKT-077. Indeed, **Ia** appears to show chemical characteristics comparable to the precursor but with a thiazole core as spacer between the flat benzothiazole terminal ring and pyridine group of the prototype, being that this one changed in a phenyl ring for compound **Ia** (see Figure 11).

**Figure 12:**
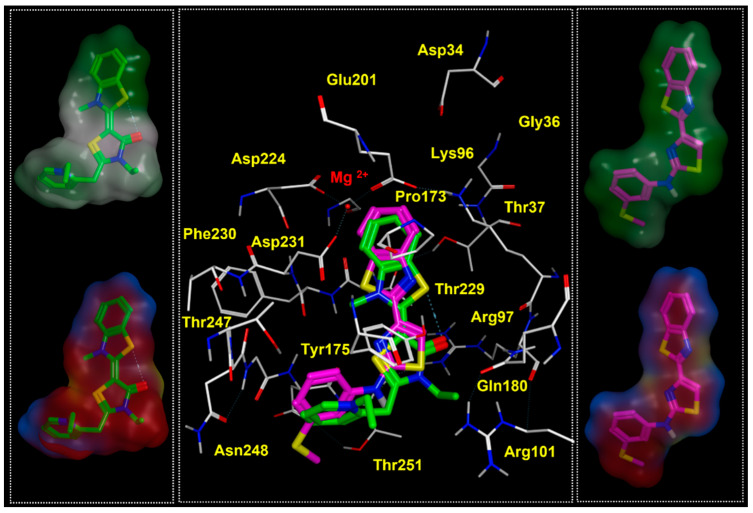
Docking positioning at the human HSP70 protein of the HSP70 inhibitor MKT-077 (C atom; green) and F508del CFTR modulator **Ia** (C atom; magenta). The related hydrophobic and electrostatic properties are shown as hydrophobic properties (**upper** side) and activeLP (**lower** side) maps onto the compound Connolly surfaces.

Our preliminary computational studies supported the structural similarity between MKT-077 and **Ia**, revealing comparable electrostatic properties and hydrophobic contact areas onto the related Connolly surface. In fact, the two compounds share similar shape and distribution of hydrophobic features, suggesting a key role played by the aromatic and hydrophobic ring within the HSP70 inhibitor chemical structure. As shown in Figure 12 (upper left and right side of the image, MKT-077 and **Ia** are reported), the extended hydrophobic area of the two derivatives is represented in green, while less lipophilic groups are highlighted in white.

Running activeLP calculations gives a perspective of the overall mild polar, H-bonding, and hydrophobic properties of the two compounds, being shown in blue, yellow, and red, respectively (see Figure 12, down, left, and right side of the image, MKT-077 and **Ia** are reported).

Thus, **Ia** was endowed by adequate electrostatic properties and H-bonding features, thanks to the thiazole ring heteroatoms, if compared to the electro-negatively charged carbonyl group of the MKT-077 thiazolidine one.

In addition, molecular docking calculations revealed a common binding positioning for both MKT-077 and **Ia** within the human HSP70 putative allosteric binding site, detecting the previously mentioned π–π stacking and cation-π contacts with the key residue Tyr175 and Mg^2+^. As shown in Figure 12, the amino-aryl moiety of **Ia** was properly superposed on the prototype pyridine ring, while the thiazole core overlapped the MKT-077 thiazolidine ring. This allowed compound **Ia** to maintain the necessary Van der Waals and polar contacts with the surrounding residues Gly249, Tyr175, Arg97, and Arg101. On the other hand, the bicyclic ring of both the two compounds was engaged in cation-p stacking with the metal ion and Lys96.

In order to better explore the effectiveness of aromatic features, enriched with electro withdrawing substituents, aimed to improve the polar contacts with their neighboring residues, also we consider two **Ia** analogues, namely **IIa** and **IIIa** (see chemical structures in Figure 11).

In particular, the presence of the disubstituted phenyl ring at the amino-aryl portion (**IIa**) or tethered to the position 4 of the thiazole core (**IIIa**) is expected to simulate the behavior played by the benzothiazole of MKT-077.

Accordingly, based on the docking positioning of **IIa** and **IIIa**, the disubstituted phenyl ring of the two compounds quite overlapped the benzothiophene-based ring of MKT-077 (see Figures S4 and S5). In this way, **II**–**IIIa** maintained the required cation- stacking with the metal ion, while the terminal mono-substituted aromatic ring guarantees the proper π–π interactions with Tyr175.

Based on these encouraging data, the aforementioned compounds **I**–**IIIa** have been submitted to the following chemical synthesis and biological evaluation. In particular, we synthesized compounds **I**–**IIa** based on the chemical synthesis previously described [39], and then measured their cellular effects as pharmacological chaperones, in combination with correctors VX-809 and **7m**.

### 2.5. Biological Evaluation of I–IIIa in Presence of F508del CFTR Correctors

Compounds **I**–**IIIa** had been synthesized based on the chemical synthesis previously described [39], and then, by exploiting the YFP-based functional assay, we performed a preliminary evaluation of their efficacy in rescuing mutant F508del-CFTR trafficking, as single agents or in combination with correctors VX-809 and **7m [41]**.

In order to assess the effect of HSP70 pharmacological inhibition on mutant CFTR rescue, we first evaluated the ability of our reference compound MKT-077 to improve CFTR trafficking and function, when administered in the presence of VX-809. To this aim, we exploited the YFP-based functional assay.

**Figure 13:**
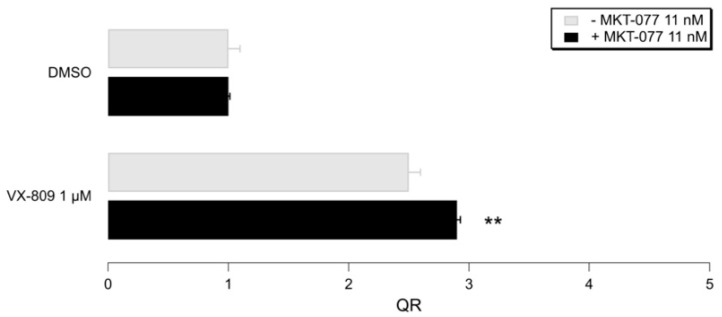
Representative bar graphs showing F508del-CFTR activity, measured with the HS-YFP microplate reader assay. CFBE41o- cells were treated for 24h, with the indicated compounds. Data in the graph are reported as quenching rate (QR) of HS-YFP fluorescence. Data are expressed as mean ± SD, *n* = 4. Asterisks indicate statistical significance versus respective control (VX-809 in the absence of MKT-077); ** *p* < 0.01.

Our results show that cells treated with VX809 displayed an increase in iodide influx that was further enhanced when MKT-077 (10nM) was added in combination with VX809. As shown in Figure 13, these data are in agreement with the data reported in the literature [31].

We then focused on the MKT-077 highly related analogue **Ia**. As in the case of MKT-077, the YFP-based functional assay demonstrated its rescue activity, as well as its comparable additive effect, when co-administered with VX-809. Interestingly, these data strongly support: (i) the common indirect CFTR modulator ability likely via HSP70 inhibition and then (ii) the effectiveness of neutral substituents at terminal aromatic ring (such as the compound **Ia** phenyl ring). Indeed, biological assays reported in the literature also demonstrated the maintained anti-proliferative activity of derivatives bearing neutral pyridines, targeting the HSP70 protein [55].

**Figure 14:**
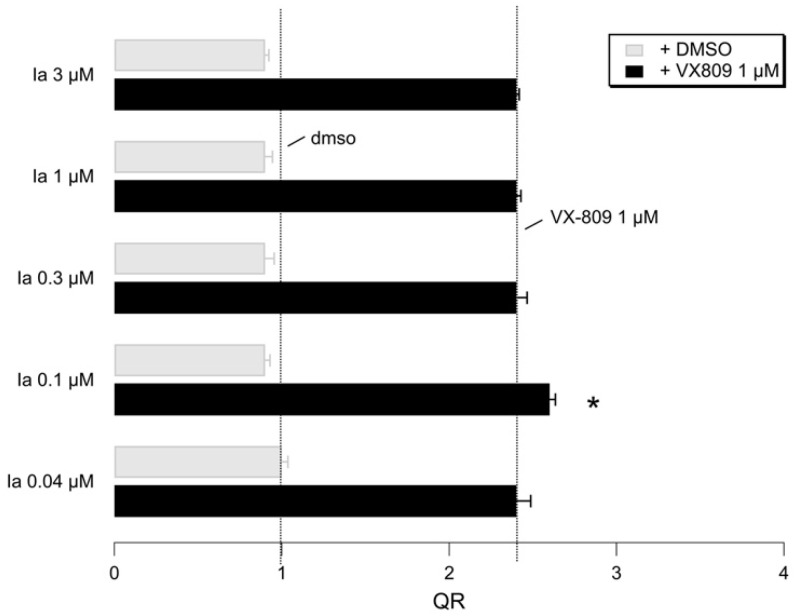
Representative bar graphs showing F508del-CFTR activity measured with the HS-YFP microplate reader assay. CFBE41o- cells were treated for 24h with indicated compounds. Data in the graph are reported as the quenching rate (QR) of HS-YFP fluorescence. Data are expressed as mean ± SD, *n* = 4. Asterisk indicates statistical significance versus VX-809 1 µM alone; * *p* < 0.05.

Similar additive effects of **Ia** were also observed, when combined with another corrector, the in-house VX-809 hybrid **7m** (see Figure 14).

**Figure 15:**
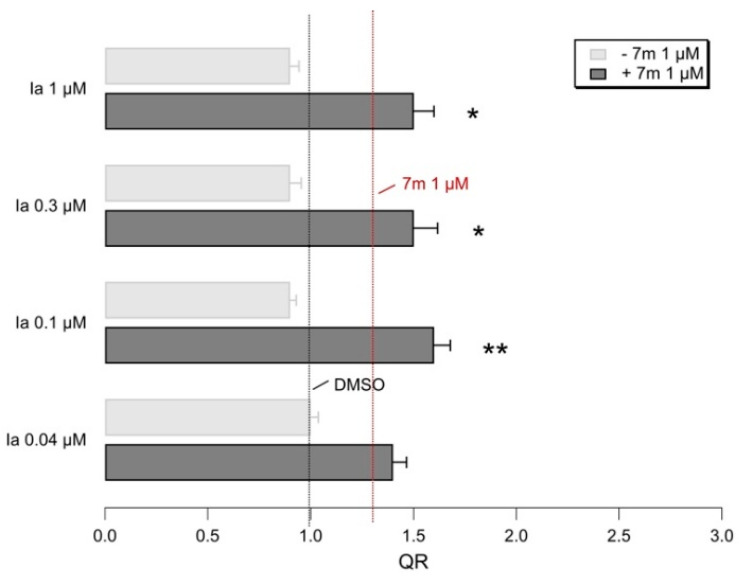
Representative bar graphs showing F508del-CFTR activity, measured with the HS-YFP microplate reader assay. CFBE41o- cells were treated for 24h, with the indicated compounds. Data in the graph are reported as quenching rate (QR) of HS-YFP fluorescence. Data are expressed as mean ± SD, *n* = 4. Asterisks indicate statistical significance versus respective control: * *p* < 0.05; ** *p* < 0.01.

In Figure 15, the thiazole **Ia** improves the activity of **7m** when used at nanomolar concentrations, similar to what happened with the VX-809.

**Figure 16:**
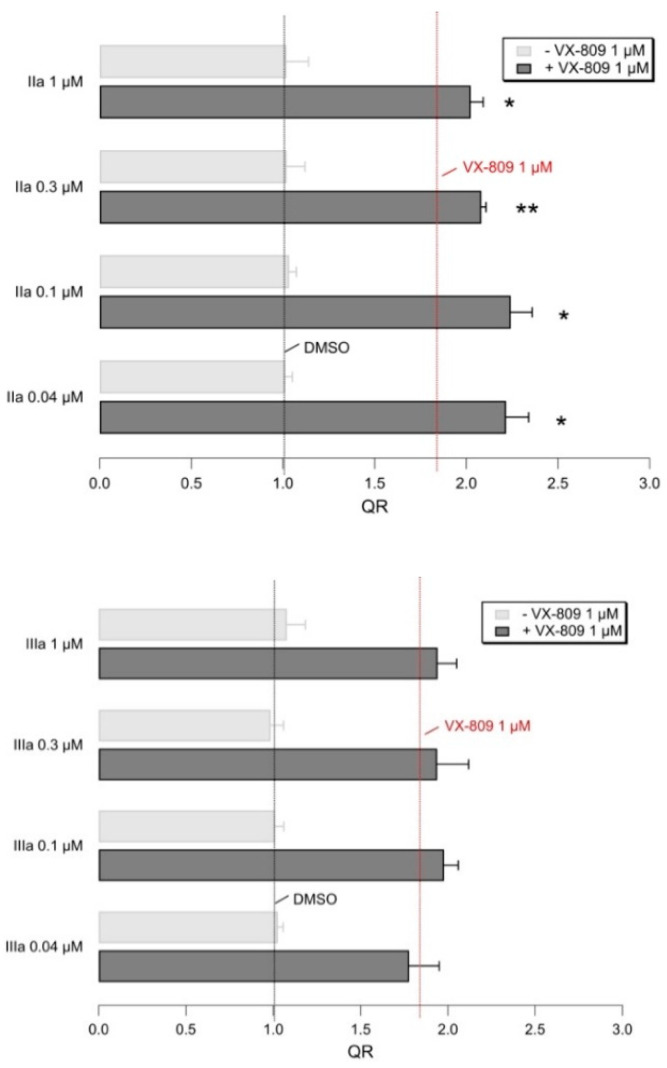
Representative bar graphs showing F508del-CFTR activity, measured with the HS-YFP microplate reader assay. CFBE41o- cells were treated for 24 h, with the indicated compounds. Data in the graph are reported as quenching rate (QR) of HS-YFP fluorescence. Data are expressed as mean ± SD, *n* = 4. Asterisks indicate statistical significance versus respective control (VX-809 1 µM alone): * *p* < 0.05; ** *p* < 0.01.

As regarding compounds **II**–**IIIa, II**a was the most effective, able to enhance the F508del CFTR rescue ability of VX-809, as previously described for the reference MKT-077 and benzothiazole-containing AAT **Ia** (see Figure 16). **II**a was effective at comparable concentrations, with respect to the previous **Ia** (0.1 μM).

Administering **II**–**IIIa**, in the presence of the hybrid **7m**, also allowed us to modestly improve the compound corrector ability at low micromolar concentration (0.3 μM), as reported in Figure S6.

Further analyses of compound efficacy and mechanisms of action as CFTR modulators are needed to characterize these compounds, including testing in more relevant cell models, such as primary human bronchial cells from F508del-CFTR homozygous patients. These analyses are, however, beyond the scope of the present study.

### 2.6. In Silico PK Profile Prediction

Recently, the drug discovery process relied on the predictive ability of in-silico evaluation of absorption, distribution, metabolism, and excretion parameters (ADME). Evaluation by computational methods of the putative pharmacokinetic (PK) properties of ligands, deeply accelerated the hit-to-lead and lead optimization process [56,57,58]. In the search of effective and drug-like HSP70 modulators, it should be noticed that MKT-077 was found to be susceptible to rapid metabolism, due to oxidation of the benzothiazole ring and of the pyridinium group [55]. The following efforts had been attempted to design putative more drug-like analogues, leading to YM-01 (lacking any cation portion) and substituted benzothiazole-based compounds, such as JG98 and further congeners (JG231 and JG345) [37], being JG231 the most promising.

Thus, herein we explored in silico the predicted main PK properties, in terms of the drug-like profile of I-IIIa here discussed, in comparison with those calculated for the reference compound MKT-077 and previously mentioned highly related analogues YM-01, JG98, JG231, and JG345.

Initially, we considered any putative violation of the well-known Lipinski’ rule [59] and Veber’ rule [60], proceeding with calculation of the logarithmic ratio of the octanol–water partitioning coefficient (cLogP), molecular weight (MW) of derivatives, H-bonding acceptor number (HBA), donor groups (HBD), number of rotatable bonds (nRot_bond), and topological polar surface area (TPSA), as well as of total polar surface area (ASA_P) (see Table 5).

It is well-known that the first ones (Lipinski’s rule) suggest for ligands exhibiting MW < 500, cLogP < 5, HBA < 10, and HBD < 5, while the rule described by Veber concerns drug bioavailability, thanks to nRot_bonds ≤ 10, the sum of HBA and HBD < 12, and the TPSA descriptor ≤ 140 Å^2^.

According to the pK properties prediction, all the compounds fulfil Lipinski’s and Veber’s rules, with the exception of the suggested MW and cLogP values, which were quite > 500 for JG231, JG345 and >5 for **II**–**IIIa**, respectively. On the other hand, all the reported AATs experienced adequate molecular weight values (MW < 500), if compared to the MKT-077 analogues JG231 and JG345 (MW > 500).

Then, the in silico prediction of ADME properties was extended to human intestinal absorption (HIA), volume of distribution (Vd), and the evaluation of the plasmatic protein binding event (%PPB), as well as of the ligand affinity toward human serum albumin (LogKa HSA). These calculations had been performed, in order to assess the putative oral bioavailability as a percentage (%F) (see Table 6).

As shown in Table 6, while the MKT-077 analogues exhibiting a cation portion (YM-01, JG98, JG231, and JG345) are predicted as poorly absorbed (HIA% = 2–51%), with the exception of JG231 (HIA= 74%), the three proposed AATs **I**–**IIIa** are endowed by optimal HIA percentage values (100%). Even if **I**–**IIIa** displayed comparable plasmatic protein binding values (%PPB = 99.59–99.74%), with respect to JG231 (%PPB = 98.10%), they are characterized by higher bioavailability values (%F = 38.2–83.9%), with **Ia** being the most interesting (%F= 83.9).

In addition, **I**–**IIIa** featured ameliorated human serum albumin binding affinity (logKa HSA = 4.91–5.23) and volume of distribution (V =4.2–4.8 l/Kg) values, rather than JG231 (logKa HSA = 3.61; V = 5.1), which was designed as optimized analogue of MKT-077.

## 3. Material and Methods

### 3.1. Ligand Preparation

All the studied correctors were manually built by the MOE Builder program [45] and then were parametrized (AM1 partial charges as calculation method), and energy was minimized by the energy minimize program, using a MMFF94x forcefield and RMS (root mean square) equal to 0.0001 Kcal/mol/A^2^ of the MOE compute module, to produce a single low-energy conformation for each ligand [45]

### 3.2. Protein Preparation and In Silico Analysis

Both the two selected human and bovine HSP70 X-ray data had been collected from the protein data bank [38] and explored, thanks to the previous cited MOE software [45].

Alignment and superimposition of the two proteins have been performed by MOE. The related protein align module implements a modified version of the alignment methodology, originally introduced into molecular biology by [61]. In this approach, alignments are computed by optimizing a function based on residue similarity scores (obtained from applying an amino acid substitution matrix to pairs of aligned residues) and gap penalties. Penalties are imposed for introducing and extending gaps in one sequence, with respect to another. The final optimized function value is referred to as the alignment score. The basis of sequence-only alignment is the amino acid substitution matrix. MOE includes matrices derived directly from families of aligned proteins, as well as matrices derived by application of an evolutionary model to a set of closely related matrices. These matrices reside in a library included in the MOE software [45].

Calculation of the molecular electrostatic, hydrophobic, and ActiveLP properties at the Connolly surface of the human HSP70 protein had been performed, thanks to the surface and map tool implemented in MOE [45]. In particular, specific electrostatic properties had been reported onto the molecular surface of the protein, as preferred locations of hydrophobic, H-bond acceptor, and H-bond donor sites, based on the solutions of the Poisson–Boltzmann equation.

The molecular surface represents an approximation to the solvent-excluded surface, previously mentioned as the Connolly surface. The solvent-excluded surface encloses the volume, from which a probe sphere (usually with water radius 1.4 Å) is excluded when it rolls over a molecule. If the atoms of the molecule are represented as spheres having van der Waals radii, then the solvent-excluded volume comprises these sphere volumes, plus the regions in-between that are too small for the probe to fit into [62]. The surfaces of those regions, between neighboring atoms, are smooth concavities and are sometimes referred to as re-entrant [63,64]. The solvent-excluded surface is related to the accessible surface, which is the surface traced out by the center of a probe sphere rolling over the atoms of the molecule. In contrast to the re-entrant surface, the accessible surface has sharp valleys, where the probe surface touches the van der Waals spheres of two or more atoms.

An analytical method to calculate the solvent-excluded surface was first described in [65], and other methods have appeared over the years [64]. MOE uses the method of level sets, as proposed by [66].

### 3.3. Molecular Docking Studies

All the molecular docking calculations, herein described, had been performed by means of the DOCK tool implemented in MOE [45], choosing, as a binding site, the one identified thanks to the MOE site finder module, agreeing with the one described in the literature, regarding the bovine HSP70 and MKT-077 putative complex. Specific details of the docking module were previously reported [56]. Briefly, the alpha triangle, as a placement algorithm, was chosen, running by superposition of compound atom triplets and triplets of biological target site points. The enzyme points are shown as alpha sphere centers. At each iteration, a random conformation is chosen. A random triplet of compound atoms and a random triplet of alpha sphere centers are exploited to determine the pose. After calculation of the enthalpy-based affinity DG scoring parameter, the proposed best-scored fifty poses had been refined by the induced fit method to the final ten ones. These poses were rescored, considering the alpha HB methodology, based on H-bonding estimation.

This affinity DG function estimates the enthalpic contribution to the free energy of binding, by means of a linear function reported in our previous work, as well as details about the induced fit module [67].

### 3.4. QSAR Analysis

QSAR studies were performed based on calculations of about three hundred molecular descriptors, including 2D and 3D parameters, by means of the MOE software. The 2D molecular descriptors are defined to be numerical properties that can be calculated from the connection table representation of a molecule (e.g., elements, formal charges, and bonds, but not atomic coordinates). The 2D descriptors are, therefore, not dependent on the conformation of a molecule and are most suitable for large database studies. They include descriptors related to physical properties, subdivided surface areas, atom and bond counts, connectivity-based descriptors, partial charges descriptors, pharmacophore features descriptors, and the so-called adjacency and distance matrix descriptors. The 3D-descriptors consist of potential energy descriptors, MOPAC descriptors, surface area, volume and shape descriptors, and conformation dependent charge descriptors.

A final data matrix of 92 rows (molecules) and 333 columns (molecular descriptors) was obtained.

As a first step, the CHECK module implemented in the chemometric package PARVUS [68] was used in order to eliminate descriptors with constant or almost constant values for all molecules. Also, pairs of variables with a correlation coefficient greater than 0.99 were removed from the data matrix.

The remaining data was collected in an *n* × m matrix, where *n* = 92 and m = 294 were the numbers of molecules and descriptors, respectively.

Then, the Kennard–Stone duplex design [53] was used for splitting the input data into a training and a test set. In this way, both sets scattered over the whole range of the considered space, defined by the descriptors of molecular structure (X) and response (y) value (pEC_50_). The Kennard–Stone duplex algorithm was applied using the first eight principal components of the autoscaled data, accounting for the 80% of the total variance. The 72 representative molecules were selected for the training set, and 20 were assigned to the test set (20% of the total molecules).

Among the 294 molecular descriptors, the most informative ones were identified using a multivariate variable selection method. In particular, iterative stepwise elimination PLS (ISEPLS) [54] was applied, in order to evaluate the relevance of the predictors, with regard to the possibility of predicting the response variable y (pEC_50_). ISE is based on the importance of the predictors, defined as:

(2) $$z_{v}=\frac{\left|b_{v}\right|s_{v}}{\sum_{v=1}^{V}\left|b_{v}\right|s_{v}}$$

where *b_v_* is the regression coefficient and *s_v_* the standard deviation of the descriptor *v*. In each elimination cycle, the descriptor with the minimum importance is eliminated, and the model is computed again with the remaining predictors.

The final model, that with the maximum predictive ability in cross validation, retained eight relevant descriptors.

QSAR was performed by the application of partial least-squares (PLS) regression analysis, considering the selected eight molecular descriptors as independent variables and corrector pEC_50_ values, as dependent variables. The leave-one-out validation procedure was used to check the internal predictability of the derived models. The predictive ability of the derived model was finally evaluated for the test set compounds (expressed as r^2^_pred_), by using the following equation:

r^2^_pred_ = (SD − PRESS)/SD (3)

SD is the sum of the squared deviations between the biological activities of the test set molecules and the mean activity of the training set compounds, and PRESS is the sum of the squared deviation between the observed and predicted activities of the test set compounds.

### 3.5. In Silico Evaluation ADME Properties

The prediction of any parameter related to ADME properties was performed, thanks to the Advanced Chemistry Development (ACD) Percepta platform [69]. This software works on the basis of the implemented training libraries, which include different series of compounds, in tandem with their experimentally evaluated pharmacokinetic properties.

### 3.6. Biological Evaluation

#### 3.6.1. Cell Culture

The bronchial epithelial CFBE41o- cells stably co-expressing F508del-CFTR and the halide-sensitive yellow fluorescent protein (HS-YFP) were cultured in MEM medium, supplemented with 10% fetal calf serum, 2 mM L-glutamine, 100 U/mL penicillin, and 100 mg/mL streptomycin.

#### 3.6.2. Fluorescence Assay for CFTR Activity

CFBE41o-cells with stable expression of mutant CFTR and HS-YFP were plated on clear-bottom, 96-well black microplates (Corning Life Sciences, Acton, MA, USA), at a density of 50,000 cells/well and kept at 37 °C in 5% CO_2_ for 24 h.

For the corrector assay, CFBE41o-cells were treated for a further 24 h, with compounds and/or VX-809. After treatment, the culture medium was removed, and cells in each well were stimulated for 30 min at 37 °C with 60 mL PBS (containing 137 mM NaCl, 2.7 mM KCl, 8.1 mM Na_2_HPO_4_, 1.5 mM KH_2_PO_4_, 1mM CaCl_2_, and 0.5 mM MgCl_2_), plus forskolin (20 µM) and genistein (50 µM).

Prior to the assay, CFBE41o- cell plates were transferred to a microplate’s reader (FluoStar Galaxy; BMG Labtech, Offenburg, Germany), equipped with high quality excitation (HQ500/20X: 500 ± 10 nm) and emission (HQ535/30M: 535 ± 15 nm) filters for YFP (Chroma Technology, Brattleboro, VT). Fluorescence was monitored continuously for 14 s, with 2 s before and 12 s after injection of an iodide-substituted solution (165 µL of a modified PBS, containing I- instead of Cl^-^; final I^-^ concentration in the well: 100 mM). Data were normalized to the initial background-subtracted fluorescence. To determine the fluorescence quenching rate, associated with I- influx, the final 10 s of data for each well were fitted with an exponential function to extrapolate initial slope (dF/dt).

Dose-response relationships from each experiment were fitted with the Hill equation, using the Igor software (WaveMetrics) to calculate EC_50_, maximal effect, and Hill coefficient.

## 4. Conclusions

The design of compounds targeting further biological targets involved in the CFTR physiological pathway, such as the molecular chaperone Hsp70, was found to be a promising strategy to optimize drug combinations for the treatment of CF. Accordingly, the allosteric HSP70 inhibitor MKT-077 proved to deeply amplify the corrector ability of VX-809. Despite this, it was withdrawn by Phase I clinical trials because of poor metabolic stability.

In this work, we reported, for the first time, structure-based studies related to the putative docking mode of MKT-077, as well as of several analogues described in the literature, considering the recent X-ray crystallographic structure of the human HSP70. This allowed us to point out the main interaction supporting their HSP70 allosteric inhibitor ability, including cation-π and π–π stacking, with the conserve residue Tyr175. All these compounds had been further explored by means QSAR analysis and used as reference compounds for the in-silico evaluation of AATs (**I**–**IIIa**), as putative HSP70 inhibitors, to be exploited in combination with VX-809. Following biological assays confirmed the effectiveness of **I**–**IIIa** in the presence of the reference F508del CFTR corrector VX-809, with **IIa** being the most promising. Further assays, performed in combination with the hybrid **7m**, confirmed the indirect CFTR modulator ability featured by these AATs, when co-administered in the presence of potent F508del CFTR correctors. As a result, the biological assays validated the computational approach, herein proposed to guide the search of novel putative human Hsp70 inhibitors. Furthermore, prediction of the **I**–**IIIa** ADME and physicochemical properties versus MKT-077 and related optimized analogues (such as JG-231) enlightened favorable characteristics for the proposed AATs. This is expected to pave the way for the rational design of novel compounds lacking pyridinium ring. This could turn into new derivatives, endowed with a better bioavailability and metabolic stability.

Further analyses are, however, required to confirm the efficacy of putative HSP70 inhibitors, in order to improve mutant CFTR rescue, when co-administered with CFTR modulators. The development of compounds able to maximize CFTR function, when co-administered with modulator drugs, is of great interest, in particular for those patients bearing mutations displaying trafficking and/or gating defects poorly responsive to already approved modulators.

Overall, the positive outcome derived from this study verified the reliability of the applied computational studies, as well as the applied virtual screening protocol, shedding light on the thiazole core, as a privileged scaffold for the design of optimized CFTR modulators, probably via inhibition of the HSP70 protein.

## Figures and Tables

**Table 1:** Comparison of the putative MKT-077 binding domain at the bovine HSP70 cavity and human HSP70. The key residues for the ligand binding, based on mutagenesis experiments, are labelled in cyan [37].

Protein PDB Code	Binding Site: Pairwise Amino Acids						
3HSC	K71	R72	V82	P147	Y149	F150	T222
6CZ1	K96	R97	S107	P173	Y175	F176	T247
3HSC	A223	G224	D225	T226	H227	L228	
6CZ1	N248	G249	D250	T251	H252	L263	

**Table 2:** List of the selected descriptors, in tandem with the related type, series, and relative importance index (RI).

Descriptor	Type	Series	RI
bpol	Sum of the absolute value of the difference between atomic polarizabilities of all bonded atoms in the molecule (including implicit hydrogens).	2D-I ^a^	0.293554
SlogP_VSA1	Sum of *v_i_*, such that *L_i_* is in −0.4, −0.2.	2D-II ^b^	0.320719
SlogP_VSA3	Sum of *v_i_*, such that *L_i_* is in (0,0.1).	2D-II ^b^	0.138811
Q_VSA_POS	Total positive van der Waals surface area. This is the sum of the *vi*, such that *q_i_* is non-negative. The vi are calculated using a connection table approximation.	2D-V	0.112977
vsa_hyd	Approximation to the sum of VDW surface areas of hydrophobic atoms (Å^2^).	2D-VI	0.289878
E_str	Bond stretch potential energy.	3D-I	1.000000
E_strain	Local strain energy: the current energy minus the value of the energy at a near local minimum.	3D-I	0.726455
vsurf_DD23	Contact distances of vsurf_DDmin (three descriptors).	3D-IV ^c^	0.148377

^a^ CRC Handbook of Chemistry and Physics; CRC Press (1994). ^b^ The subdivided surface areas are descriptors, based on an approximate accessible van der Waals surface area (in Å^2^) calculation for each atom, *v_i_* along with some other atomic property, *p_i_*. The *v_i_* are calculated using a connection table approximation. Each descriptor in a series is defined to be the sum of the *v_i_* over all atoms *i*, such that *p_i_* is in a specified range ^a,b^ *L_i_* denotes the contribution to logP (*o*/*w*) for atom *i*, as calculated in the SlogP descriptor. *R_i_* denotes the contribution to molar refractivity for atom i, as calculated in the SMR descriptor (Wildman, S.A., Crippen, G.M.; Prediction of Physiochemical Parameters by Atomic Contributions; J. Chem. Inf. Comput. Sci. 39 No. 5 (1999) 868–873). The ranges were determined by percentile subdivision over a large collection of compounds. ^c^ These descriptors depend on the structure connectivity and conformation (dimensions are measured in Å). The vsurf descriptors are similar to the VolSurf descriptors (Cruciani, G., Crivori, P., Carrupt, P.-A., Testa, B.; Molecular Fields in Quantitative Structure-Permeation Relationships: the VolSurf Approach; J. Mol. Struct. (Theochem) 503 (2000) 17–30).

**Table 3:** List of the mean values of the most relevant 3D and 2D descriptors featured by pyridine- and thiazole-based MKT-077 analogues.

MCF-7 Inhibitors	E_Str_mean_	SlogP_VSA1_mean_	bpol_mean_	Q_VSA_POS_mean_	Vsa_hyd_mean_	pEC_50_
Pyridine-based MKT-077 analogues	18.4938	9.0545	34.7501	167.2119	361.5962	4.72–6.10
Thiazole-based MKT-077 analogues	3.5747	11.0400	42.7463	322.0256	453.5605	>6.80

**Table 4:** List of the mean values of the bpol, Q_VSA_POS, and Vsa_hyd descriptors, as featured by thiazole-based MKT-077 analogues.

MCF-7 Inhibitors	bpol_mean_	Q_VSA_POS_mean_	Vsa_hyd_mean_	pEC_50_
Five-membered ring-based MKT-077 analogues	40.9062	341.1889	436.2678	6.92–7.00
Benzyl-containing MKT-077 analogues	43.2063	317.2347	457.8837	6.82–7.32

**Table 5:** Calculated properties, related to the Lipinski’s rules and to the Veber’s rules, referred to the AATs **I**-**IIIa** and reference compounds MKT-077, YM-01, JG98, JG231, and JG345.

Compound	MW ^a^	HBA ^b^	HBD ^c^	nRot bond ^d^	TPSA ^e^	cLogP ^f^
**MKT-077**	396.56	1	0	3	27.43	−0.88
**YM-01**	382.53	1	0	2	27.43	−1.31
**JG98**	499.10	1	0	4	27.43	2.59
**JG231**	584.03	1	0	4	27.43	3.04
**JG345**	550.75	2	0	7	53.73	2.55
**Ia**	355.51	2	1	4	37.81	4.92
**IIa**	360.93	1	1	5	24.92	5.97
**IIIa**	367.32	1	1	4	24.92	5.70

^a^ molecular weight (MW) of compounds, ^b^ number of H-bonding acceptor, ^c^ number of donor groups, ^d^ number of rotable bonds, ^e^ topological polar surface area, ^f^ total polar surface area, cLogP represents the logarithmic ratio of the octanol–water partitioning coefficient.

**Table 6:** Calculated ADMET parameters, related to absorption and distribution properties, as referred to the AATs **I**–**IIIa** and reference compounds MKT-077, YM-01, JG98, JG231, and JG345.

Compound	HIA (%) ^a^	Vd (l/kg) ^b^	%PPB ^c^	LogKa HAS ^d^	%F (Oral) ^e^
**MKT-077**	2%	2.8	76.62	2.79	0.0
**YM-01**	3%	2.4	73.28	2.75	0.0
**JG98**	51	4.7	97.96	3.75	0.0
**JG231**	74	5.1	98.10	3.61	0.0
**JG345**	49	4.1	96.86	3.44	0.0
**Ia**	100	4.3	99.59	4.91	83.9
**IIa**	100	4.8	99.68	5.01	62.2
**IIIa**	100	4.2	99.74	5.23	38.2

^a^ HIA represents the human intestinal absorption, expressed as percentage of the molecule able to pass through the intestinal membrane; ^b^ prediction of volume of distribution (Vd) of the compound in the body; ^c^ plasmatic protein binding event; ^d^ ligand affinity toward human serum albumin; ^e^ oral bioavailability, as a percentage.

## Data Availability

Data is contained within the article and Supplementary Materials.

## Supplementary Materials

The following are available online at www.mdpi.com/article/10.3390/ph14121296/s1. Figure S1: electrostatic properties of the HSP70 inhibitor YM-01 and related ligplot at the HSP70 protein; Figure S2: molecular docking positioning of compound **43** and related ligplot; Figure S3: chemical structures of VX-809 and of compound **7m**; Figure S4: molecular docking positioning of MKT-077 and of compound **IIa**; Figure S5: molecular docking positioning of MKT-077 and of compound **IIIa**; Figure S6: representative bar graphs, showing F508del-CFTR activity, measured with the HS-YFP microplate reader assay; Table S1: chemical structure and biological data of MKT-077 and the related six-membered ring analogs; Table S2: chemical structure of the five-membered ring analogs of the HSP70 inhibitor MKT-077; Table S3: molecular docking scoring functions, as related to MKT-077 and analogues; Table S4: list of the predicted and experimental MCF-7 antiproliferative ability for all the dataset compounds, in tandem with the collected descriptors.
