# Supplementary material for: Probing Allosteric Hsp70 Inhibitors by Molecular Modelling Studies to Expedite the Development of Novel Combined F508del CFTR Modulators
Source: Pharmaceuticals (Basel). 2021 Dec 12;14(12):1296. doi: 10.3390/ph14121296 (PMC8709398; doi:10.3390/ph14121296)

SUPPLEMENTARY MATERIAL

Table S1. Chemical structure of MKT-077 and of the related six-membered ring analogs. The antiproliferative activity values in MCF-7 cell line in also reported.

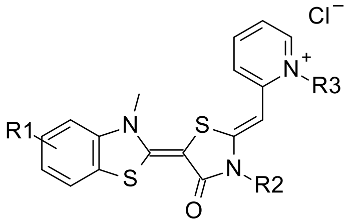

Compound	R1	R2	R3	MCF-7 pEC_{50} M
**MKT-077**	H	$-CH_2CH_3$	$-CH_2CH_3$	5.66
**YM-01**	H	$-CH_2CH_3$	$-CH_3$	5.28
**1**	3-F	$-CH_2CH_3$	$-CH_3$	5.66
**2**	4-F	$-CH_2CH_3$	$-CH_3$	4.74
**3**	5-F	$-CH_2CH_3$	$-CH_3$	5.21
**4**	6-F	$-CH_2CH_3$	$-CH_3$	5.07
**5**	3-F	$-CH_2CH_3$	$-CH_2CH_3$	6.00
**6**	4-F	$-CH_2CH_3$	$-CH_2CH_3$	5.62
**7**	5-F	$-CH_2CH_3$	$-CH_2CH_3$	6.05
**8**	6-F	$-CH_2CH_3$	$-CH_2CH_3$	6.10
**9**	3-Cl	$-CH_2CH_3$	$-CH_3$	5.11
**10**	4-Cl	$-CH_2CH_3$	$-CH_3$	4.55
**11**	5-Cl	$-CH_2CH_3$	$-CH_3$	5.02
**12**	6-Cl	$-CH_2CH_3$	$-CH_3$	5.28
**13**	$4-OCH_3$	$-CH_2CH_3$	$-CH_3$	4.85
**14**	$5-OCH_3$	$-CH_2CH_3$	$-CH_3$	4.96
**15**	$4-CF_3$	$-CH_2CH_3$	$-CH_3$	4.52

**16**	5-CF_3	-CH_2CH_3	-CH_3	4.52
**17**	H	-$CH_2CH{=}CH_2$	-CH_3	5.08
**18**	H	-CH_2Ph	-CH_3	5.54
**19**	H	-CH_2CH_3	-$CH_2CH_2CH_3$	5.55
**20**	H	-CH_2CH_3	-CH_2Ph	5.82
**21**	H	-CH_2CH_3	-CH_2CH_2OH	5.16
**22**	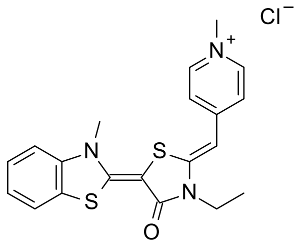			4.72
**23**	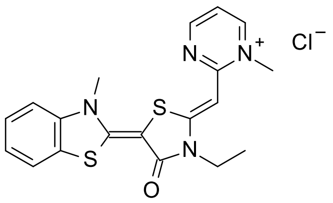			5.04

Table S2. Chemical structure of the five-membered ring analogs of the HSP70 inhibitor MKT-077. The antiproliferative activity values in MCF-7 cell line in also reported.

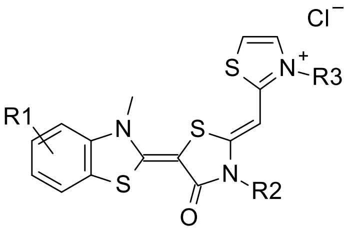

**Compound**	**R1**	**R2**	**R3**	**MCF-7 pEC_{50} M**
**24**	H	-CH_2CH_3	-CH_3	6.15
**25**	H	-CH_2CH_3	-CH_2CH_3	6.10
**26**	H	-CH_2CH_3	-CH_2Ph	6.22
**27 (JG-83)**	3-F	-CH_2CH_3	-CH_2Ph	6.00
**28 (JG-84)**	3-Cl	-CH_2CH_3	-CH_2Ph	6.40
**29**	4-Cl	-CH_2CH_3	-CH_2Ph	6.22
**30 (JG-98)**	5-Cl	-CH_2CH_3	-CH_2Ph	6.40
**31**	6-Cl	-CH_2CH_3	-CH_2Ph	6.30
**32**	5-Br	-CH_2CH_3	-CH_2Ph	6.36
**33**	4-Br	-CH_2CH_3	-CH_2Ph	6.57
**34**	5-NO_2	-CH_2CH_3	-CH_2Ph	5.30
**35**	4-SO_2CH_3	-CH_2CH_3	-CH_2Ph	5.30
**36**	5-CF_3	-CH_2CH_3	-CH_2Ph	6.28
**37**	4-OCF_3	-CH_2CH_3	-CH_2Ph	6.59
**38**	5-OCF_3	-CH_2CH_3	-CH_2Ph	6.40
**39**	4-CH_3	-CH_2CH_3	-CH_2Ph	6.68
**40 (JG-194)**	5-CH_3	-CH_2CH_3	-CH_2Ph	6.80
**41**	3-CH_3	-CH_2CH_3	-CH_2Ph	6.60
**42**	6-CH_3	-CH_2CH_3	-CH_2Ph	6.54
**43**	5-CH_2CH_3	-CH_2CH_3	-CH_2Ph	7.10
**44**	4-OCH_3	-CH_2CH_3	-CH_2Ph	6.43
**45**	5-OCH_3	-CH_2CH_3	-CH_2Ph	6.89
**46**	5-SCH_3	-CH_2CH_3	-CH_2Ph	6.89
**47**	5-Cl	-$CH_2CH=CH_2$	-CH_2Ph	6.77
**48**	5-Cl	*c*-Pr	-CH_2Ph	6.48
**49**	5-Cl	-CH_2CH_3	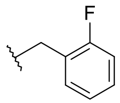	6.80
**50**	5-Cl	-CH_2CH_3	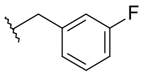	6.32

**51**	5-Cl	-CH_2CH_3	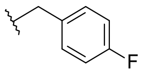	6.21
**52**	5-Cl	-CH_2CH_3	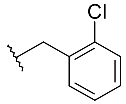	6.72
**53**	5-Cl	-CH_2CH_3	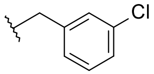	6.59
**54**	5-Cl	-CH_2CH_3	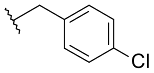	5.84
**55**	5-Cl	-CH_2CH_3	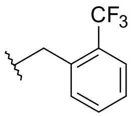	6.89
**56**	5-Cl	-CH_2CH_3	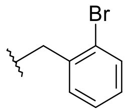	6.72
**57**	5-Cl	-CH_2CH_3	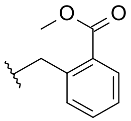	6.77
**58**	5-Cl	-CH_2CH_3	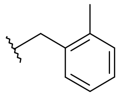	6.72
**59**	5-Cl	-CH_2CH_3	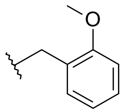	6.40
**60**	5-Cl	-CH_2CH_3	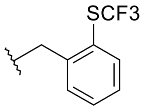	6.89

**61**	5-Cl	$-CH_2CH_3$	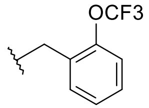	6.66
**62 (JG-258)**	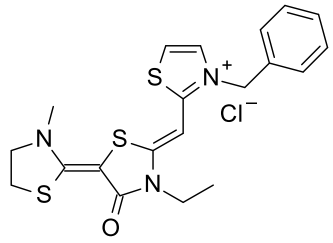			5.30
**63**	5-Cl	$-CH_2CH_3$	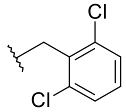	6.80
**64**	5-Cl	$-CH_2CH_3$	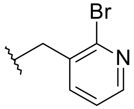	5.82
**65**	5-Cl	$-CH_2CH_3$	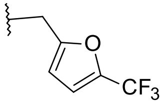	6.68
**66**	5-Cl	$-CH_2CH_3$	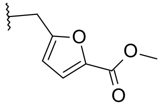	5.82
**67**	5-Cl	$-CH_2CH_3$	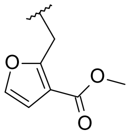	6.09
**68**	5-Cl	$-CH_2CH_3$	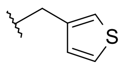	6.14
**69**	5-Cl	$-CH_2CH_3$	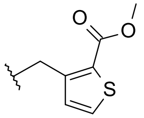	6.80

**70**	5-Cl	-CH_2CH_3	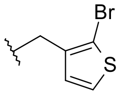	6.28
**71 (JG-231)**	5-Cl	-CH_2CH_3	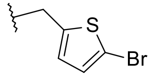	6.92
**72**	5-Cl	-CH_2CH_3	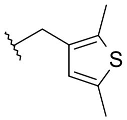	7.01
**73**	5-Cl	-CH_2CH_3	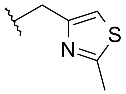	6.02
**74**	5-Cl	-CH_2CH_3	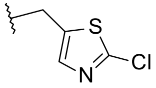	5.30
**75**	5-Cl	-CH_2CH_3	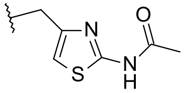	5.30
**76**	5-Cl	-CH_2CH_3	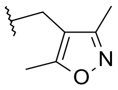	5.30
**77**	4-CH_3	-CH_2CH_3	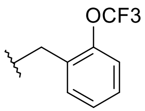	7.05
**78**	5-CH_3	-CH_2CH_3	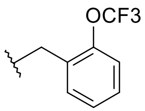	7.19
**79**	4-OCH_3	-CH_2CH_3	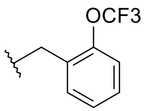	6.80

**80**	5-OCH_3	-CH_2CH_3	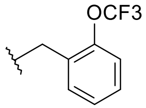	7.15
**81**	5-*i*Pr	-CH_2CH_3	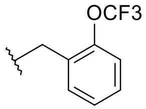	7.05
**82**	5-OCH_3	-CH_2CH_3	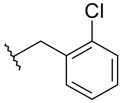	7.32
**83**	5-SCH_3	-CH_2CH_3	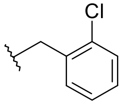	6.82
**84**	5-OCH_3	-CH_2CH_3	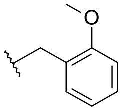	7.12
**85**	5-CH_3	-CH_2CH_3	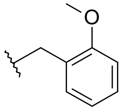	7.15
**86 (JG-345)**	5-CH_2CH_3	-CH_2CH_3	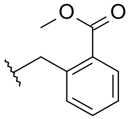	7.31
**87**	4-CH_3	-CH_2CH_3	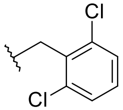	7.06
**88**	5-CH_2CH_3	-CH_2CH_3	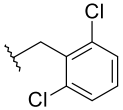	7.03

**89 (JG-294)**	5-Br	$-CH_2CH_3$	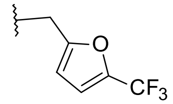	7.00
**90**	$5-CH_2CH_3$	$-CH_2CH_3$	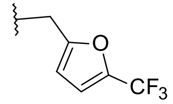	6.96

Table S3. Molecular docking scoring functions related to the performed calculations about compounds MKT-077, YM-01 and **1**-**90**. The derived docking poses were prioritized by the score values of the lowest energy pose of the compounds docked to the protein structure, as follows: S: the final score, which is the score of the last stage of refinement; E_conf: the energy of the conformer. If there is a refinement stage, this is the energy calculated at the end of the refinement; E_place: score from the placement stage; E_score1 and E_score2 score from rescoring stages 1 and 2; E_refine: score from the refinement stage, calculated to be the sum of the van der Waals electrostatics and solvation energies, under the Generalized Born solvation model (GB/VI) [65].

Compound	S	E_conf	E_place	E_score1	E_refine	E_score2
**mkt077**	-101.3389	110.1202	-15.1308	-74.0109	88.8863	-101.3389
**ym01**	-103.7933	105.6025	-15.8583	-87.2654	71.4362	-103.7933
**1**	-89.7909	115.5620	-19.0678	-77.4590	29.9912	-89.7909
**2**	-102.9088	100.8143	-15.6220	-88.9346	68.3339	-102.9088
**3**	-112.5449	109.8237	-16.8267	-71.6729	87.2043	-112.5449
**4**	-102.6369	97.3167	-17.6441	-58.4592	81.9538	-102.6369
**5**	-100.4820	121.8921	-14.1693	-64.2847	47.7577	-100.4820
**6**	-96.2329	106.3949	-19.8044	-75.6559	45.6973	-96.2329
**7**	-97.5354	115.6799	-19.8800	-59.4245	45.7081	-97.5354
**8**	-102.8838	103.2593	-19.4467	-56.0534	29.9829	-102.8838
**9**	-104.4358	112.5641	-13.2229	-68.3148	93.3679	-104.4358
**10**	-95.2572	102.9637	-16.1564	-75.3014	47.6932	-95.2572
**11**	-96.5341	104.6809	-16.0752	-71.2779	18.6010	-96.5341

**12**	-94.8027	101.8098	-15.1614	-70.6595	56.8228	-94.8027
**13**	-97.4615	100.7749	-19.6532	-66.2685	36.9735	-97.4615
**14**	-111.2127	111.5197	-15.6530	-66.7264	106.4995	-111.2127
**15**	-106.9431	114.4750	-18.7323	-71.0306	56.8261	-106.9431
**16**	-100.1619	109.5814	-17.2746	-87.0941	66.8203	-100.1619
**17**	-103.9539	107.2442	-14.6121	-73.4579	71.5607	-103.9539
**18**	-105.7882	113.3393	-20.7369	-76.6678	96.0211	-105.7882
**19**	-98.2356	114.9143	-17.6284	-77.4850	54.0188	-98.2356
**20**	-107.6419	116.2076	-12.0728	-58.8231	131.8117	-107.6419
**21**	-110.3339	115.4353	-14.6959	-82.8606	51.9426	-110.3339
**22**	-106.7224	99.9257	-15.0486	-70.5223	14.7444	-106.7224
**23**	-99.8901	119.2580	-14.1416	-72.1079	73.2788	-99.8901
**24**	-100.8400	109.8640	-15.0318	-72.5402	62.9795	-100.8400
**25**	-101.0749	114.7133	-13.3639	-73.6967	30.1692	-101.0749
**26**	-86.6006	118.9158	-12.9532	-45.8892	40.7572	-86.6006
**27 (JG83)**	-108.8426	128.4486	-10.9725	-54.5266	141.0714	-108.8426
**28 (JG84)**	-104.2601	125.1533	-8.2088	-40.6036	173.1362	-104.2601
**29**	-91.7560	116.1619	-8.9935	-45.8307	92.8109	-91.7560
**30 (JG98)**	-112.7902	118.5010	-9.1000	-49.5762	126.4644	-112.7902
**31**	-96.1231	115.1728	-12.6998	-60.8062	70.3151	-96.1231
**32**	-105.1347	88.4065	-17.6163	-48.6824	32.0778	-105.1347
**33**	-110.8819	90.5092	-18.0488	-72.3901	46.9365	-110.8819
**34**	-105.4230	95.1217	-14.1239	-61.0107	36.6077	-105.4230
**35**	-109.6560	99.0282	-12.7976	-58.1562	74.1193	-109.6560
**36**	-109.0624	92.3788	-15.8586	-82.7240	18.7429	-109.0624
**37**	-111.1659	84.3841	-14.3260	-57.9252	32.8707	-111.1659
**38**	-121.9380	94.5506	-21.2172	-76.6891	60.5865	-121.9380
**39**	-101.4917	84.9356	-15.2221	-51.9676	43.6149	-101.4917
**40 (JG-194)**	-103.0400	88.1796	-19.3014	-42.8709	44.2028	-103.0400
**41**	-107.2420	94.3644	-16.4418	-51.7908	59.4595	-107.2420
**42**	-105.0742	80.2745	-18.8937	-63.3199	47.3902	-105.0742
**43**	-101.8727	90.9977	-16.8632	-63.9228	29.3012	-101.8727

**44**	-111.1275	83.5577	-16.3113	-72.8035	59.2620	-111.1275
**45**	-109.7775	93.6380	-15.3164	-61.6533	33.5859	-109.7775
**46**	-103.1466	87.3704	-15.8315	-50.8868	48.9941	-103.1466
**47**	-120.3074	90.1872	-13.8089	-47.1378	22.6933	-120.3074
**48**	-115.3868	176.9884	-13.2850	-40.7007	84.0253	-115.3868
**49**	-108.7866	90.3885	-16.6638	-51.3304	27.1359	-108.7866
**50**	-106.2646	90.6003	-17.1603	-53.2316	35.8413	-106.2646
**51**	-105.3420	81.2371	-13.5123	-43.3172	73.8398	-105.3420
**52**	-110.5340	78.8374	-12.6215	-58.1161	63.3255	-110.5340
**53**	-99.0479	77.4058	-19.7797	-80.4350	25.5430	-99.0479
**54**	-90.5044	77.4290	-15.2329	-51.7368	28.5519	-90.5044
**55**	-143.6963	82.6628	-19.9439	-90.4292	43.9055	-143.6963
**56**	-132.7871	79.4997	-19.8431	-86.5885	32.8556	-132.7871
**57**	-162.6770	83.4680	-20.4291	-116.8257	38.3305	-162.6770
**58**	-128.1816	79.5421	-16.7062	-69.6853	29.7603	-128.1816
**59**	-145.3857	81.4468	-14.9998	-61.7339	33.6795	-145.3857
**60**	-145.4450	82.8753	-21.6035	-94.7113	41.2778	-145.4450
**61**	-151.0686	83.5910	-17.0874	-76.4066	37.8341	-151.0686
**62 (JG 258)**	-103.4759	66.2657	-16.0246	-57.4951	63.4346	-103.4759
**63**	-120.9056	78.3748	-12.2454	-59.1150	50.3108	-120.9056
**64**	-114.7239	77.5900	-16.7651	-70.8452	25.9040	-114.7239
**65**	-111.8042	110.7218	-20.5833	-65.0187	26.8879	-111.8042
**66**	-103.7390	111.1897	-13.4220	-61.8679	28.8077	-103.7390
**67**	-124.9913	116.7311	-21.7255	-101.2431	52.3298	-124.9913
**68**	-111.4148	96.4950	-14.6940	-72.8119	44.1871	-111.4148
**69**	-118.5379	101.6310	-17.2674	-76.8882	15.6891	-118.5379
**70**	-108.3175	97.2885	-19.8916	-79.4882	25.8893	-108.3175
**71 (JG 231)**	-112.5995	96.0632	-19.5211	-86.2182	23.0074	-112.5995
**72**	-101.6321	96.0571	-15.0300	-56.2239	48.9005	-101.6321
**73**	-118.4933	92.6373	-21.1978	-71.5936	22.6071	-118.4933
**74**	-114.1580	79.9425	-22.5384	-77.4950	39.5277	-114.1580
**75**	-118.8434	98.1689	-12.9653	-61.7393	89.0002	-118.8434

**76**	-114.7829	102.1778	-15.5580	-79.7464	39.4429	-114.7829
**77**	-88.5994	87.1570	-14.6017	-48.2235	48.7905	-88.5994
**78**	-96.7157	87.1787	-16.0771	-48.0514	152.6839	-96.7157
**79**	-90.7484	90.2318	-13.8857	-65.9145	57.8928	-90.7484
**80**	-101.3302	90.1710	-13.8371	-53.5047	56.2865	-101.3302
**81**	-95.1307	88.5257	-11.7004	-42.5547	160.8540	-95.1307
**82**	-108.7697	86.7023	-15.8427	-47.2247	44.3320	-108.7697
**83**	-104.0375	84.8388	-20.3797	-46.7463	50.5775	-104.0375
**84**	-94.4717	88.3288	-15.3398	-62.6696	193.8054	-94.4717
**85**	-103.6242	85.4243	-15.7049	-62.3091	47.5505	-103.6242
**86 (JG345)**	-101.9287	87.5901	-15.2252	-65.4852	51.4096	-101.9287
**87**	-95.1809	85.0116	-16.4385	-44.8516	45.0311	-95.1809
**88**	-96.9606	85.0346	-13.7356	-51.3036	31.3457	-96.9606
**89 (JG294)**	-107.5412	110.2276	-15.9689	-56.7933	5.3447	-107.5412
**90**	-103.5457	111.3143	-18.2141	-73.6590	0.9481	-103.5457

Figure S1. Electrostatic properties of the HSP70 inhibitor YM-01 shown onto the related molecular surface (left side). The corresponding ligplot is depicted on the right.

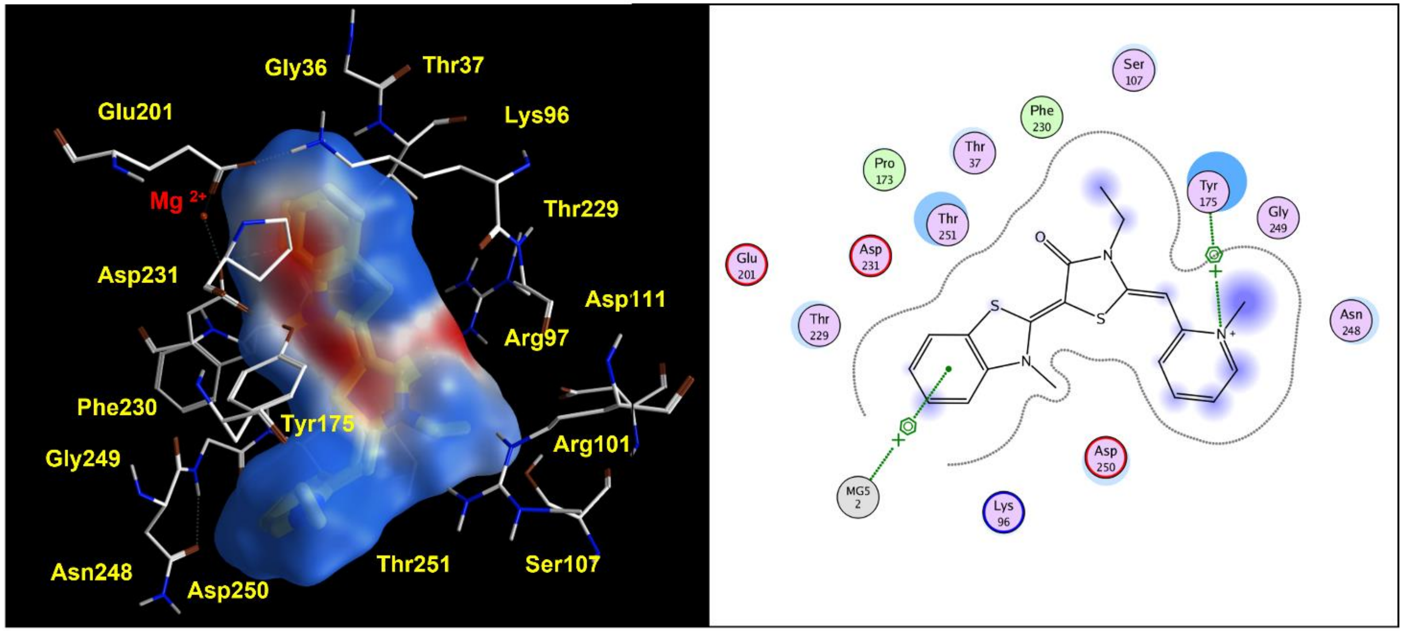

Figure S2. Docking positioning of **43** (C atom; magenta) within the modelled human HSP70 protein. The most relevant residues are shown in yellow (left side), while the key contact involving the conserved residue Tyr175 is highlighted in the corresponding ligplot (right side).

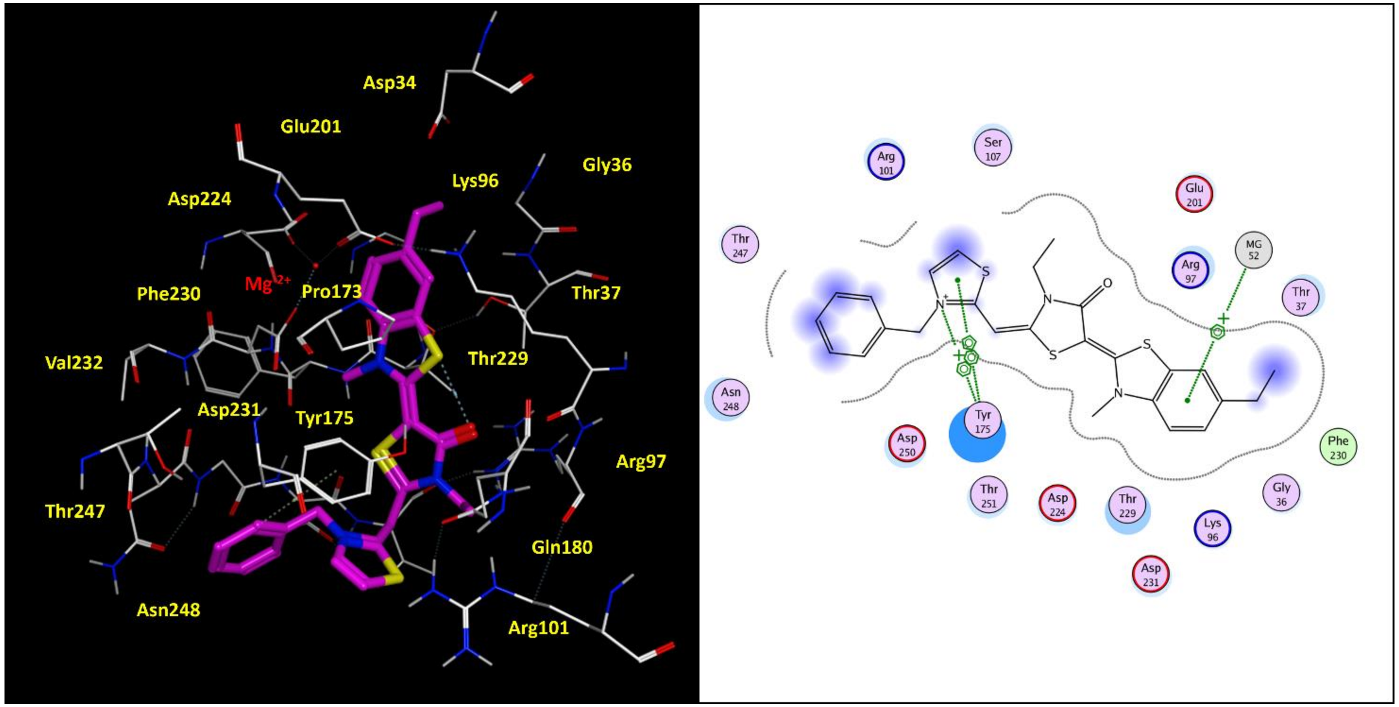

Table S4. The predicted (Pred. pEC_{50}) and experimental MCF-7 (Exp. pEC_{50}) antiproliferative ability for all the compounds are reported in tandem with the collected descriptors. The compounds included in the test set are underlined along the first column.

Compound	Exp. pEC_{50}	bpol	E_str	E_strain	Q_VSA_POS	SlogP_VSA1	SlogP_VSA3	vsa_hyd	vsurf_DD23	Pred. pEC_{50}
**mkt077**	5.66	35.5086	18.4482	34.8344	159.7084	8.3831	18.4396	358.7236	1.0000	5.23
**<u>ym01</u>**	5.28	33.3221	18.3849	35.6545	159.8532	8.3831	18.4396	342.9151	7.1589	5.31
**1**	5.66	33.4319	17.6715	36.2350	149.2335	8.3831	18.4396	348.0361	7.3824	5.44
**<u>2</u>**	4.74	33.4319	17.9743	35.2530	155.9330	8.3831	18.4396	348.0361	7.1589	5.37
**3**	5.21	33.4319	18.0402	35.4828	164.6087	8.3831	18.4396	348.0361	7.1589	5.38
**4**	5.07	33.4319	17.9485	35.2332	164.6087	8.3831	18.4396	348.0361	7.1589	5.39
**5**	6.00	35.6183	18.1840	37.3352	157.7643	8.3831	18.4396	363.8446	3.6056	5.49
**6**	5.62	35.6183	18.0463	35.9264	164.4638	8.3831	18.4396	363.8446	6.6521	5.60
**7**	6.05	35.6183	18.1370	36.0252	164.4638	8.3831	18.4396	363.8446	6.6521	5.59
**8**	6.10	35.6183	18.2542	36.0179	164.4638	8.3831	18.4396	363.8446	6.6521	5.58
**9**	5.11	32.6489	18.1061	37.1631	176.4942	8.3831	18.4396	361.2083	3.9370	5.33
**10**	4.55	32.6489	17.9492	35.2312	176.4942	8.3831	18.4396	361.2083	7.3824	5.43
**11**	5.02	32.6489	18.1134	35.4826	185.1698	8.3831	18.4396	361.2083	1.1180	5.17
**12**	5.28	32.6489	18.0809	35.7327	181.0139	8.3831	18.4396	361.2083	0.5000	5.15
**<u>13</u>**	4.85	37.4246	18.0581	37.1540	176.8425	8.3831	18.4396	371.2236	0.5000	5.54

**14**	4.96	37.4246	18.2463	37.7594	170.1429	8.3831	18.4396	371.2236	6.6895	5.80
**15**	4.52	35.8379	18.6205	37.1538	155.9577	8.3831	18.4396	374.0866	7.0000	5.65
**16**	4.52	35.8379	18.6485	37.2949	164.6333	8.3831	18.4396	374.0866	1.0000	5.40
**17**	5.08	33.3221	19.2116	36.0428	164.3699	8.3831	18.4396	358.7236	0.5000	5.03
**18**	5.54	35.5086	20.4108	38.0850	181.5763	8.3831	0.0000	394.7577	2.1213	5.63
**19**	5.55	37.6950	18.5525	36.4609	168.2391	8.3831	18.4396	374.5321	0.5000	5.49
**20**	5.82	37.6950	20.4776	36.8793	168.2391	8.3831	18.4396	410.5662	0.5000	5.48
**21**	5.16	35.5086	18.4517	35.4861	165.7672	8.3831	39.3658	351.3736	0.5000	4.92
**22**	4.72	33.3221	18.1639	37.1102	146.6866	8.3831	18.4396	342.9151	1.0000	5.10
**23**	5.04	33.5489	20.1658	39.3311	193.5707	25.1686	18.4396	324.8981	2.7386	4.37
**24**	6.15	33.4157	11.9704	25.2794	164.3835	8.3831	18.4396	335.0109	6.3443	5.63
**25**	6.10	35.6021	11.9714	28.5981	172.9143	8.3831	18.4396	350.8194	6.1847	5.97
**26**	6.22	37.7886	14.2671	28.2634	194.6374	8.3831	18.4396	402.6619	6.7268	6.17
**27 (JG83)**	6.00	37.8983	13.8101	28.7190	184.0177	8.3831	18.4396	407.7829	0.5000	5.99
**28 (JG84)**	6.40	37.1153	14.0420	29.3860	219.9539	8.3831	18.4396	420.9551	6.6895	6.32
**29**	6.22	37.1153	13.8927	27.7334	219.9539	8.3831	18.4396	420.9551	0.7071	6.02
**30 (JG98)**	6.40	37.1153	14.1744	28.1404	219.9539	8.3831	18.4396	420.9551	6.8374	6.27
**31**	6.30	37.1153	13.9577	28.2092	224.4737	8.3831	18.4396	420.9551	6.5192	6.29
**32**	6.36	37.9853	3.1594	0.0369	225.3212	8.3831	18.4396	432.7305	4.7170	6.62
**33**	6.57	37.9853	3.1536	0.0483	225.3212	8.3831	18.4396	432.7305	5.7663	6.66
**34**	5.30	37.9513	3.2001	0.5372	197.9793	15.4687	69.3746	397.5810	0.5000	5.28
**35**	5.30	46.4510	3.2384	1.9766	194.3058	56.5548	18.4396	422.4226	5.7228	5.35

**36**	6.28	40.3043	3.3928	0.0690	190.7419	8.3831	18.4396	433.8334	0.5000	6.52
**<u>37</u>**	6.59	42.2203	3.3137	0.0827	205.4504	18.2005	18.4396	446.3333	5.1478	6.57
**38**	6.40	42.2203	3.3143	0.0624	198.7508	18.2005	18.4396	446.3333	0.5000	6.36
**<u>39</u>**	6.68	39.9750	3.1883	0.0520	194.4925	8.3831	18.4396	418.4704	4.8990	6.63
**<u>40 (JG-194</u>)**	6.80	39.9750	3.2232	0.0446	194.4925	8.3831	18.4396	418.4704	0.5000	6.44
**41**	6.60	39.9750	3.2385	0.0507	194.4925	8.3831	18.4396	418.4704	4.8990	6.62
**42**	6.54	39.9750	3.1712	0.1094	196.7011	8.3831	18.4396	418.4704	4.8990	6.64
**43**	7.10	42.1614	3.3674	0.1074	203.0233	8.3831	18.4396	434.2789	0.5000	6.65
**44**	6.43	41.8910	3.3452	0.1256	202.9511	8.3831	18.4396	430.9704	0.8660	6.64
**45**	6.89	41.8910	3.3467	0.1445	196.2515	8.3831	18.4396	430.9704	4.5000	6.79
**<u>46</u>**	6.89	42.2550	3.3938	0.1225	207.5536	8.3831	18.4396	442.1832	5.1235	6.91
**47**	6.77	37.1153	3.0861	0.2233	211.2783	8.3831	18.4396	436.7636	0.5000	6.40
**48**	6.48	37.1153	3.1106	0.2003	211.2783	8.3831	0.0000	409.5637	0.7071	6.50
**<u>49</u>**	6.80	37.2251	3.3906	0.2264	216.0338	8.3831	18.4396	426.0761	0.5000	6.32
**50**	6.32	37.2251	3.2971	0.3959	216.0338	8.3831	18.4396	426.0761	0.5000	6.34
**51**	6.21	37.2251	3.2983	0.0053	216.0338	8.3831	18.4396	426.0761	0.5000	6.32
**52**	6.72	36.4421	3.2611	0.0058	236.5949	8.3831	18.4396	439.2483	4.8477	6.56
**53**	6.59	36.4421	3.0888	0.0055	236.5949	8.3831	18.4396	439.2483	0.5000	6.40
**54**	5.84	36.4421	3.0686	0.0058	236.5949	8.3831	18.4396	439.2483	0.5000	6.40
**<u>55</u>**	6.89	39.6311	4.0170	0.0069	216.0584	8.3831	18.4396	452.1266	7.8262	6.85
**<u>56</u>**	6.72	37.3121	3.5945	0.0070	250.6378	8.3831	18.4396	451.0237	1.1180	6.50
**57**	6.77	42.1758	3.7644	0.0071	234.5176	16.1286	18.4396	443.0136	1.4142	6.44

**58**	6.72	39.3018	3.5199	0.0062	219.8091	8.3831	18.4396	436.7636	1.0000	6.51
**59**	6.40	41.2178	3.4471	0.0070	228.2676	8.3831	18.4396	449.2636	0.5000	6.70
**60**	6.89	41.9111	3.6273	0.0048	237.7951	14.5078	18.4396	475.8393	2.2913	6.73
**61**	6.66	41.5471	3.5440	0.0062	239.4425	18.2005	18.4396	464.6265	7.8899	6.76
**62 (JG 258)**	5.30	37.7886	2.4492	0.0056	187.9378	11.5074	54.1127	366.6278	1.1180	5.53
**63**	6.80	35.7689	3.5228	0.0068	232.3295	8.3831	18.4396	457.5415	7.8102	6.72
**64**	5.82	37.5389	3.7207	0.0065	246.3724	25.1686	18.4396	437.4540	2.0616	5.83
**65**	6.68	39.3607	4.2113	0.0051	207.5277	8.3831	18.4396	416.0925	1.2247	6.32
**66**	5.82	41.9053	4.2722	0.0049	225.9868	16.1286	18.4396	406.9795	0.7071	6.13
**<u>67</u>**	6.09	41.9053	4.1194	0.0061	225.9868	16.1286	18.4396	393.3796	1.5811	6.11
**68**	6.14	37.2089	2.9807	0.0058	306.0492	8.3831	18.4396	413.0509	0.5000	6.40
**69**	6.80	42.2693	3.5565	0.0055	360.3104	16.1286	18.4396	435.1093	0.5000	6.54
**<u>70</u>**	6.28	37.4057	3.2910	0.0045	335.4059	8.3831	18.4396	443.1194	1.0000	6.60
**71 (JG 231)**	6.92	37.4057	3.0332	0.0058	335.4059	8.3831	18.4396	443.1194	0.7071	6.61
**72**	7.01	41.5818	3.0808	0.0062	340.5133	8.3831	18.4396	444.6679	0.5000	6.87
**73**	6.02	39.6221	3.3751	0.0067	321.4255	25.1686	18.4396	422.0329	1.0000	5.96
**74**	5.30	38.9957	3.0724	0.0062	324.5344	25.1686	18.4396	393.7702	0.7071	5.79
**75**	5.30	42.3333	3.4440	0.0064	361.1889	48.4382	18.4396	413.3573	0.7071	5.24
**76**	5.30	40.1246	4.2550	0.0065	327.8089	27.9797	18.4396	407.4291	0.5000	5.69
**<u>77</u>**	7.05	44.4068	3.5440	0.0062	362.8023	18.2005	18.4396	462.1418	0.7071	6.76
**78**	7.19	44.4068	3.4802	0.0066	362.8023	18.2005	18.4396	462.1418	1.1180	6.79
**79**	6.80	46.3228	3.5273	0.0056	371.5560	18.2005	18.4396	474.6418	0.7071	6.96

**80**	7.15	46.3228	3.5445	0.0077	371.5560	18.2005	18.4396	474.6418	8.1548	7.28
**81**	7.05	48.7796	3.7325	0.0063	398.6115	18.2005	18.4396	493.7588	0.7071	7.23
**82**	7.32	41.2178	3.4647	0.0060	343.6740	8.3831	18.4396	449.2636	5.5000	7.05
**83**	6.82	41.5818	3.4710	0.0069	356.6570	8.3831	18.4396	460.4764	0.5000	6.94
**84**	7.12	45.9934	3.2960	0.0066	358.3284	8.3831	18.4396	459.2788	0.5000	7.23
**85**	7.15	44.0774	3.3050	0.0083	349.5747	8.3831	18.4396	446.7789	0.7071	7.03
**86 (JG345)**	7.31	47.2218	3.6297	0.0061	388.8322	16.1286	18.4396	456.3374	7.9687	7.32
**87**	7.06	38.6286	3.7742	0.0082	352.5017	8.3831	18.4396	455.0568	6.0208	6.92
**88**	7.03	40.8150	3.7442	0.0062	369.7338	8.3831	18.4396	470.8653	0.5000	6.93
**89 (JG294)**	7.00	40.2307	4.2578	0.0053	341.8427	8.3831	18.4396	427.8679	5.4314	6.77
**90**	6.96	44.4068	4.3851	0.0074	346.9937	8.3831	18.4396	429.4163	5.4083	7.03

Figure S3. The chemical structures of the F508del CFTR corrector VX-809 and of the in-house hybrid **7m** are shown and compared, in tandem with their potency in terms of corrector ability. The maintained benzodioxole carboxamide portion is highlighted in light violet.

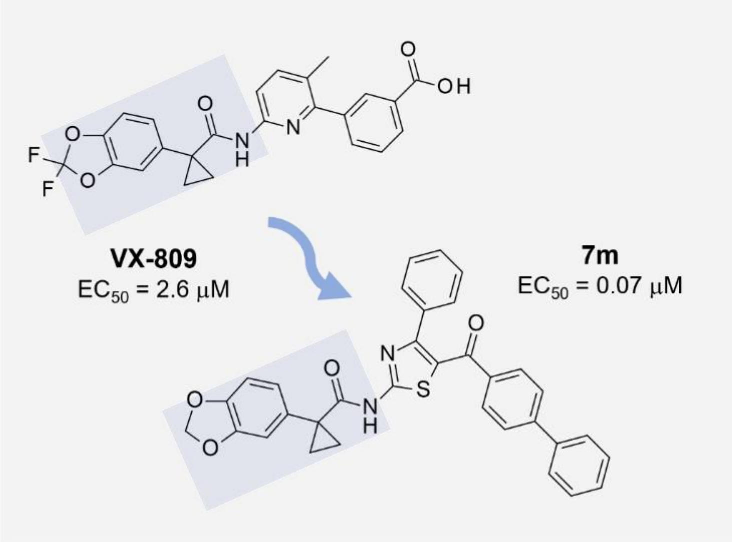

Figure S4. Docking positioning at the human HSP70 protein of the HSP70 inhibitor MKT-077 (C atom; green) and of the AAT **IIa** (C atom; magenta). The related are shown on the left.

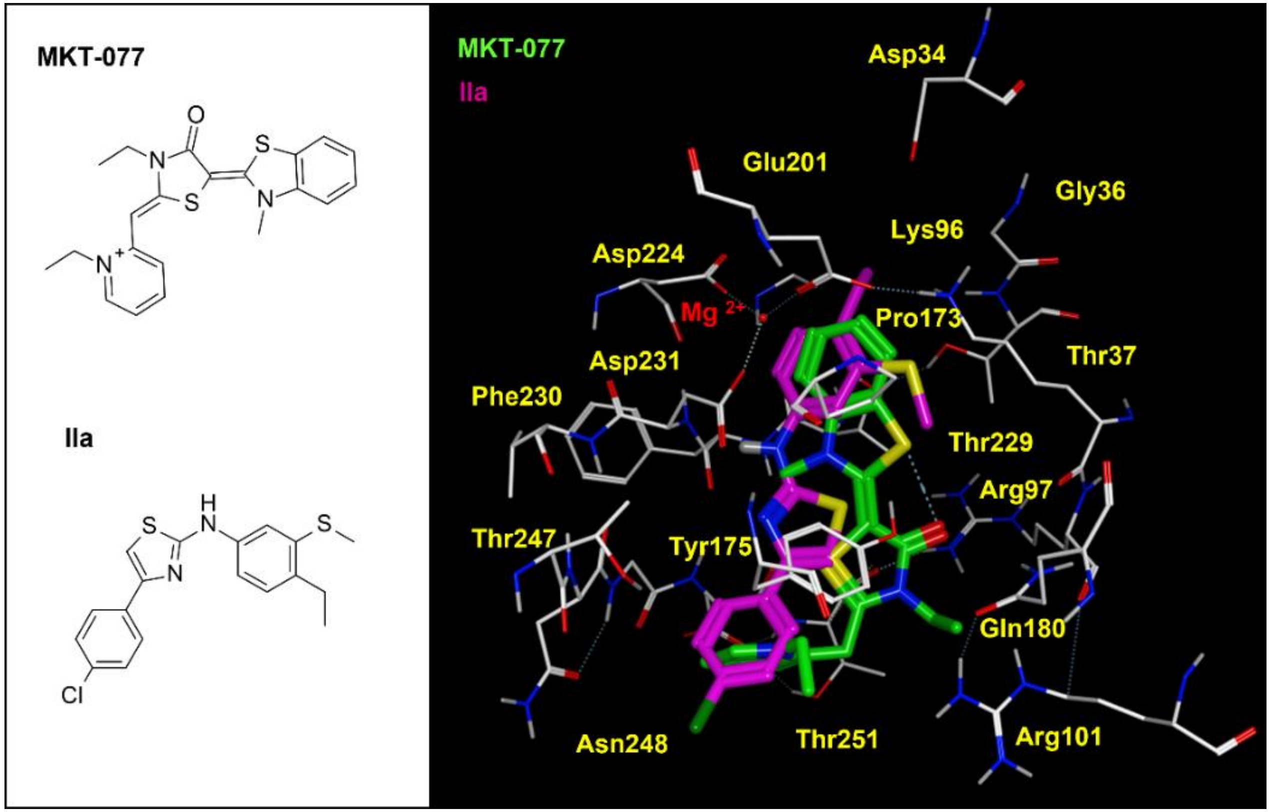

Figure S5. Docking positioning at the human HSP70 protein of the HSP70 inhibitor MKT-077 (C atom; green) and of the AAT **IIIa** (C atom; magenta). The related are shown on the left.

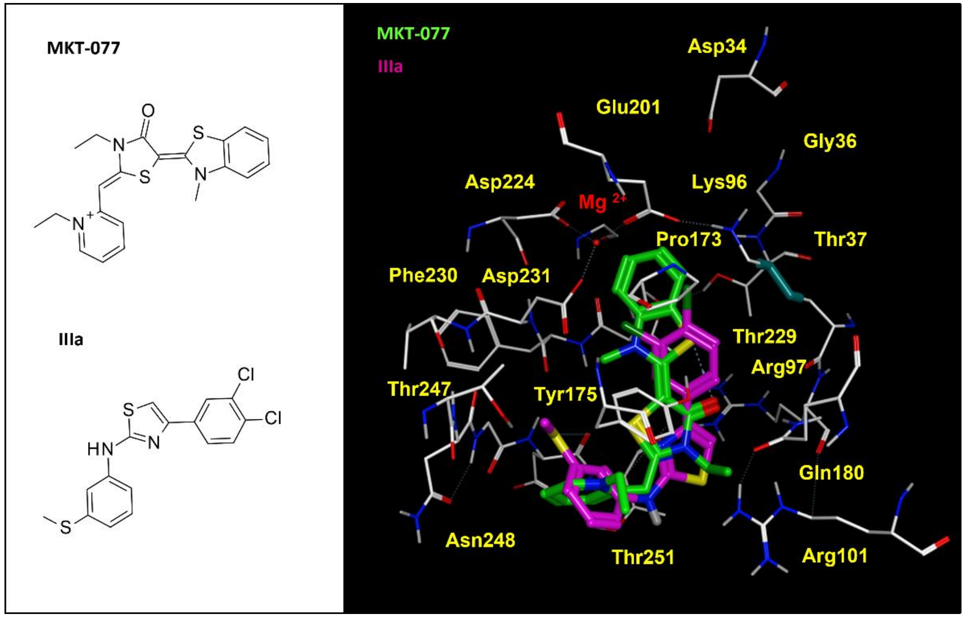

Figure S6. Representative bar graphs showing F508del-CFTR activity measured with the HS-YFP microplate reader assay. CFBE41o- cells were treated for 24h with indicated compounds. Data in the graph are reported as quenching rate (QR) of HS-YFP fluorescence. Data are expressed as mean ± SD, n = 4. Asterisks indicate statistical significance versus respective control (VX-809 1 μM alone): * P < 0.05; ** P < 0.01.

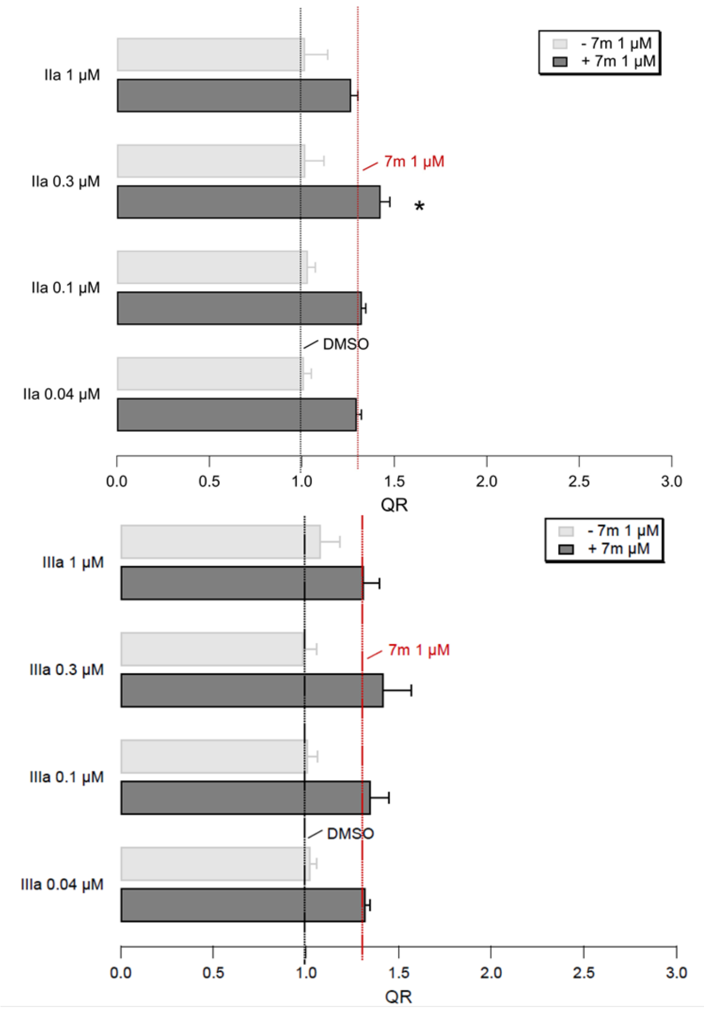

Supplement: Supplementary file 1 [file pharmaceuticals-14-01296-s001.zip › pharmaceuticals-1473768-supplementary.pdf]
